# Wound infiltrating adipocytes are not myofibroblasts

**DOI:** 10.1038/s41467-023-38591-6

**Published:** 2023-05-25

**Authors:** Shruthi Kalgudde Gopal, Ruoxuan Dai, Ania Maria Stefanska, Meshal Ansari, Jiakuan Zhao, Pushkar Ramesh, Johannes W. Bagnoli, Donovan Correa-Gallegos, Yue Lin, Simon Christ, Ilias Angelidis, Valerio Lupperger, Carsten Marr, Lindsay C. Davies, Wolfgang Enard, Hans-Günther Machens, Herbert B. Schiller, Dongsheng Jiang, Yuval Rinkevich

**Affiliations:** 1 Institute of Regenerative Biology and Medicine, Helmholtz Center Munich, Munich, Germany; 2 Institute of Lung Health and Immunity, Helmholtz Center Munich, Munich, Germany; 3 Institute of AI for Health, Helmholtz Center Munich, Munich, Germany; 4 Anthropology and Human Genomics, Faculty of Biology, Ludwig-Maximilian University Munich, Munich, Germany; 5 Department of Microbiology, Tumour and Cell Biology (MTC), Karolinska Institute, Stockholm, Sweden; 6 Department of Plastic and Hand Surgery, Klinikum rechts der Isar, School of Medicine, Technical University of Munich, Munich, Germany

**Keywords:** Gene regulatory networks, Time-lapse imaging, Cell migration, Transdifferentiation

## Abstract

The origins of wound myofibroblasts and scar tissue remains unclear, but it is assumed to involve conversion of adipocytes into myofibroblasts. Here, we directly explore the potential plasticity of adipocytes and fibroblasts after skin injury. Using genetic lineage tracing and live imaging in explants and in wounded animals, we observe that injury induces a transient migratory state in adipocytes with vastly distinct cell migration patterns and behaviours from fibroblasts. Furthermore, migratory adipocytes, do not contribute to scar formation and remain non-fibrogenic in vitro, in vivo and upon transplantation into wounds in animals. Using single-cell and bulk transcriptomics we confirm that wound adipocytes do not convert into fibrogenic myofibroblasts. In summary, the injury-induced migratory adipocytes remain lineage-restricted and do not converge or reprogram into a fibrosing phenotype. These findings broadly impact basic and translational strategies in the regenerative medicine field, including clinical interventions for wound repair, diabetes, and fibrotic pathologies.

## Abstract (web summary)

Adipocyte plasticity during injury repair is debated. Here, the authors find that injury causes temporary adipocyte migration, but without lineage conversion to myofibroblasts. Distinct migration patterns, transcriptomics and ECM depositions indicate that adipocytes, unlike fibroblasts, do not contribute to fibrotic scars.

## Introduction

Cells undergo a gradual stepwise restrictive specification during embryonic development, acquiring lineage-specific differentiation fates to become specialized adult cell types. This gradual segregation of cellular potential during development is thought to be restrictive and maintained into, and throughout, adulthood^1^. However, many studies have challenged this notion, and called lineage-restriction into question, by proposing that fully committed cells can respond to tissue challenges, such as injury, disease or aging, by acquiring new fates. This phenotypic adaptability is termed plasticity.

The prototypical example of plasticity is the terminally differentiated stromal mesenchymal cell^2^. Mesenchyme includes mature adipocytes and fibroblasts, which have been proposed to cross-convert between lineages in response to tissue challenges, in skin and in internal organs of both mice and humans^3–11^. The impact of lineage interplay between adipocytes and fibroblasts is evident clinically in numerous disorders including diabetes, where complications associated with the disease include chronic wound healing and fibrotic pathologies such as renal disease and diabetic retinopathy^12^.

Lineage interplay between adipocytes and fibroblasts is also evident in fibrotic induction, irrespective of organ. Loss of dermal adipose tissue and adipogenicity in favour of expanding stromal fibroblasts responsible for ECM deposition are consistent features of fibrosis in both mice and men. This is exemplified in skin biopsies from patients with systemic sclerosis^13^. Similar imbalances in the adipocyte: fibroblast stromal environment are seen in obese or diabetic patients undergoing fibro-inflammation in their adipose tissue, where adipocyte numbers decrease in favour of fibroblasts, a transition that is associated with fibrosis and scar formation within the connective tissues^14,15^.

The relevance of changing cellular ratios and phenotypes of fibroblasts and adipocytes within organ systems extends far beyond diabetes and fibrotic disease however. Increased frailty and deterioration in organ function have been directly linked to changes in adipose and fibroblast communities with age^16,17^. There is a clear need for further delineation of each cell lineage’s role in tissue homoeostasis and repair.

Three main hypotheses have been proposed as to how cells may transition between lineages: de-differentiation (and re-differentiation), (direct) trans-differentiation and heterotypic cell fusion^18^. (i) De-differentiation is where a cell loses its lineage-specific differentiation state and reverses into a more immature cellular state, sometimes associated with a multi-lineage differentiation potential, as seen in adult stem/progenitor cells. (ii) Trans-differentiation is the transition from one lineage to another either directly or indirectly i.e., de-differentiation followed by subsequent re-differentiation. (iii) The third conversion mechanism, heterotopic cell fusion, involves merging of two terminally differentiated and functionally distinct cell types. This form of fusion results in the formation of a hybrid cell with the combined functions of both precursor cells, and has been shown to occur in low frequency during injury^19^.

Most reported observations of interchangeability between fibroblasts and adipocytes have been based on one cell type acquiring markers associated with the other, e.g., fibroblasts expressing Perilipin-1 (*Plin1*), or adipocytes expressing alpha smooth muscle actin (*Acta2*). However, these markers are insufficient to establish the identity categorically^20,21^. Designating plasticity has equally relied on morphological changes, for example between round and lipidated adipocytes to flat bipolar fibroblasts. However, phenotype can also be deceptive, as mature adult adipocytes can shed their lipid content under certain metabolic conditions making them even harder to distinguish from fibroblasts. Such fluidity in the homoeostatic properties of adipocytes means that a more in-depth characterization of the molecular and cytostructural changes involved in response to injury is needed to definitively demonstrate cellular plasticity.

Cellular identity is not just a construct of markers and morphology. Formal proof of physiological plasticity requires the observation of a defined mature cell losing its cellular identity and functions, and metamorphosing into a distinctly different cellular identity with new functions. Such changes are, in the main, inherently achieved through transcriptional regulation, characterized by the loss of mature differentiation markers (as for de-differentiation), the expression of immature cell markers and acquisition of a new transcriptional landscape (as for trans-differentiation), supporting the attainment of these new functional properties^22^.

Here, we directly explore the plasticity of fibroblasts and white adipocytes in skin by closely analysing various transcriptomic, phenotypic, and functional criteria across these two cellular lineages. Using a combination of genetic lineage-tracing strategies, single-cell transcriptomics, live-cell imaging and tracking, transplantation assays and in vivo injury models, we study stepwise transcriptional and functional responses in mature adipocytes and fibroblasts after skin injury. We conclude that adipocytes remain lineage-restricted in response to tissue injury, and do not dedifferentiate, fuse with, or convert to myofibroblasts. Adipocytes respond to wounding stimuli, experience shifts in their motility and functionality, and play active but distinct roles from fibroblasts in the endogenous tissue healing response.

## Results

### Adipocytes retain their cell lineage identities in a skin explant model

**Fig. 1:**
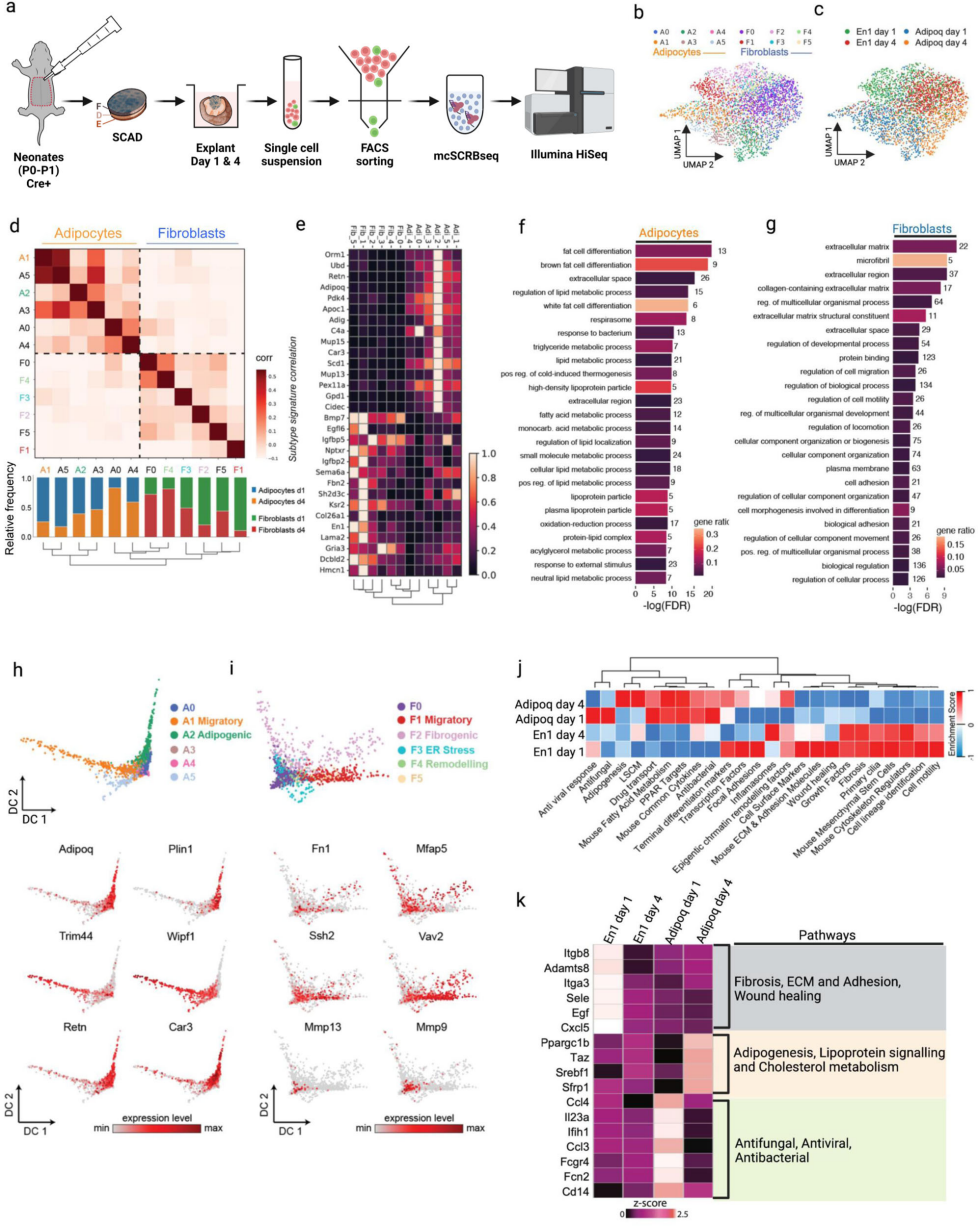
Adipocyte and fibroblast lineages retain their identities in skin explant model. **a** Schematic workflow of ex vivo whole-skin explant assay and molecular crowding single cell RNA barcoding and sequencing (mcSCRBseq). The skin explants from neonatal Adipoq^Cre^;R26^mTmG^ or En1^Cre^;R26^mTmG^ skin were cultured in 96-well plate with fascia side face up. The GFP^+^ cells were FACS sorted from explants that were collected 1 day or 4 days after culture for single cell sequencing. F, fascia; D, dermis; E, epidermis. **b** Dimension-reduced single cell transcriptomic data is visualized through Uniform Manifold Approximation and Projection (UMAP), coloured by Louvain cluster and **c** time point of extraction. **d** Similarities of marker gene signatures for the 12 cell states (6 states per lineage) along with relative frequency of each cell state per time point. Colour indicates Pearson correlation coefficients for each pairwise comparison across transcriptional cell states in adipocyte and fibroblast lineages. **e** The heatmap shows relative expression of the indicated genes across cell states and lineages. **f** Gene set enrichment results in an adipocyte core signature gene list (88 genes). **g** Gene set enrichment results in a fibroblast core signature gene list (198 genes). **h** Diffusion maps show adipocyte cell states and the gene expression levels of the indicated genes. **i** Diffusion maps show fibroblast cell states and gene expression levels of the indicated genes. **j** Pathway focused gene expression analysis of adipocytes and fibroblasts at day 1 and day 4. **k** Expression of feature genes of listed pathway in adipocytes and fibroblasts at day 1 and day 4. Z score of individual gene was normalization read counts across samples.

To address a potential phenotypic convergence of adipocyte and fibroblast lineages we performed single-cell RNA-seq (scRNA-seq) experiments. To track fibroblasts in the back-skin, we employed an En1^Cre^ reporter that labels dermal fibroblasts, and to a small extent adipocytes. The vast majority of En1-lineage cells termed Engrailed-lineage positive fibroblasts (EPFs) have been demonstrated to be the fibrogenic cell lineage responsible for scar formation in the back-skin^23–25^. Mature adipocytes were selectively tracked using an adiponectin lineage-specific reporter (Adipoq^Cre^), a hormone involved in regulating glucose levels and fatty acid breakdown, and a key biomolecule for anti-diabetic clinical interventions^26^. The two lineage-specific transgenic lines were individually crossed to a double-colour fluorescent reporter (R26^mTmG^), thereby marking mature adipocytes and fibrogenic cells with membrane-bound green fluorescent protein (GFP) in two separate transgenic reporter mice (Supplementary Fig. 1a). Skin explants were harvested from both adipocyte (Adipoq^Cre^;R26^mTmG^) and fibroblast (En1^Cre^;R26^mTmG^) double-transgenic reporter mice, cultured for up to 5 days, followed by purification of adipocytes and fibroblasts for scRNA-seq (Fig. 1a)^27–29^.

In two-dimensional uniform manifold approximation and projection (UMAP) embeddings of the single-cell transcriptomes, the two lineages largely separated but with partial overlap (Fig. 1b). The overlap was mostly observed on day 4 after culture (Fig. 1c). To characterize the heterogeneity of cell states within the two lineage-labelled populations of single cells we used Louvain clustering analysis. *Engrailed-1* marks a very early embryonic population of cells in the dermomyotome, primarily of fibroblasts but also a subset of adipocyte progenitors (APs). Indeed, two clusters of APs (cluster 1 and 9) were identified in the En1-lineage population (Supplementary Fig. 1b, c) in neonatal skin of the explant model. They were excluded in the analyses to enable mature adipocytes alone to be studied for potential cellular conversion into fibroblasts. We observed six distinct sub-clusters of cells in each of the Adipoq^Cre^ and the En1^Cre^ lineages (Fig. 1, Supplementary Fig. 1d, e). All twelve cluster identities have highly distinct marker genes and distinct enriched gene categories (Supplementary Fig. 1h, Supplementary Data 1). Next, we used Pearson’s correlation co-efficiency and measured the magnitude of relationships between the twelve clusters (Fig. 1d). This analysis indicated that the twelve clusters did not overlap, or converge, and remained separate. Despite signature variability within lineages, the two lineages stayed distinct and clustered apart under all experimental conditions.

These data indicated that adipocyte and fibroblast lineage signatures are not lost in the explants that mimic wounding and scarring. We identified a core set of 88 definitive adipocyte genes and 198 definitive fibroblast genes that remained specific to both lineages in the explants (Fig. 1e, Supplementary Fig. 1i, Supplementary Data 2). Gene ontology (GO) gene set enrichment analysis confirmed that the core set of adipocyte genes was significantly enriched for terms such as “fat cell differentiation” and “lipid metabolism” (Fig. 1f), while the fibroblast core gene set was significantly enriched for extracellular matrix genes (Fig. 1g). To further address this lineage restriction at the pathway level, we scored transcriptomic signatures of both adipocytes and fibroblasts against a selected set of pathway gene lists taken from the RT^2^ profiler PCR array (n = 84). We found that fibroblast signatures were enriched for fibrogenically active pathways like ECM and adhesion molecules, fibrosis, wound healing, focal adhesions, etc. Whereas, adipocytes were enriched for adipogenic pathways such as fatty acid metabolism, Lipoprotein signalling and cholesterol metabolism (*Lscm*), Peroxisome proliferator-activated receptor gamma (*PparƔ*) targets etc.

The adipocyte core signature contained genes such as Pyruvate dehydrogenase lipoamide kinase isozyme 4 (*Pdk4*), a gene involved in reactive oxygen species (ROS) production, which has a vital role acting as a second messenger recruiting immune cells, as well as defensive against invading bacteria at the site of injury^30,31^. Core adipocyte markers also included Orosomucoid 1 (*Orm1*) known to be immunomodulatory, Stearoyl Coa desaturase 1 (*Scd1*) which is involved in fatty acid biogenesis and Serum amyloid A 3 (*Saa3*), which is responsible for acute phase response and is induced by pro-inflammatory stimuli^32^.

Fibroblast core signature genes included Bone morphogenetic protein 7 (*Bmp7*), which is likely involved in ECM degradation, Insulin like growth factor binding protein 5 (*Igfbp5*) involved in ECM production promoting fibrosis^33^, and Nerve growth factor (*Ngf*), known to promote myofibroblast differentiation^34,35^. This indicated to us that definitive adipogenic and fibrogenic lineage markers are not lost in explants during culture (as assumed in de-differentiation), nor are they re-acquired in alternative lineages (as assumed in trans-differentiation).

Next, we focused on the individually clustering differentiation trajectories of adipocytes and fibroblasts in explants. Mature adipocytes (cluster A2) were characterized by high metabolic activity, and lipid biogenesis i.e., by expressing the Complement factor d (*Cfd*), Adiponectin (*Adipoq*), Perilipin1 (*Plin1*), and Resistin (*Retn*) genes (Supplementary Fig. 1h). Interestingly, adipocyte cluster A1 was enriched for a gene signature consistent with a migratory phenotype, embellished with active cytoskeletal remodelling, including increased expression of Was/Wasl interacting protein family member 1 (*Wipf1*), Tripartite motif containing 44 (*Trim44*), Opticin (*Optc*), and NLR family apoptosis inhibitory protein 6 (*Naip6*). It was this adipogenic cluster that appeared transcriptionally closest to fibroblasts (Fig. 1h). Still, migratory adipocytes (cluster A1) could be consistently distinguished from fibroblast subpopulations based on their expression of adipogenic markers such as *Cfd* (Supplementary Fig. 1f), an adipokine involved in cell signalling and insulin secretion, and *Cidec* (Supplementary Fig. 1g), a gene encoding cell death activator CIDE-3 and involved in lipid droplet enlargement. Together, these data indicated that mature adipocytes might undergo rearrangement and active remodelling of cytoskeletal actin filaments in explants, reminiscent of a migratory mesenchymal cell, while still retaining the definitive adipogenic-lineage markers and not acquiring the definitive fibrogenic-cell markers.

Analysis of cell states of En1-lineage positive cells identified enrichment for several different biological processes (Fig. 1i). Fibroblast cluster F1 was enriched for cell migration associated genes such as Vav guanine nucleotide exchange factor 2 (*Vav2*), Slingshot protein phosphatase 2 (*Ssh2*), Myosin light chain kinase (*Mylk*), Unc-51 like kinase 4 (*Ulk4*), IQ motif containing GTPase activating protein 1 (*Iqgap1*), and was transcriptionally the closest fibroblast cluster to the migratory adipocyte cluster. We identified a state consistent with fibrogenic gene expression (cluster F2), including Collagen type 1 alpha 1 chain (*Col1a1*), Collagen type 3 alpha 1 chain (*Col3a1*), Fibronectin (*Fn1*), Periostin (*Postn*), Decorin (*Dcn*), Microfibril associated protein 5 (*Mfap5*). Cluster F3 demonstrated a genotypic profile central to endoplasmic reticulum (ER) stress processes with expression of Mesemcephalic astrocyte derived neurotrophic factor (*Manf*), Cysteine rich with EGF like domains 2 (*Creld2*), Heat shock protein 90 beta family member 1 (*Hsp90b1*). Cluster F4 however, was enriched for ECM remodelling genes and processes, expressing Matrix metallopeptidases 9 and 13 (*Mmp9, Mmp13*), Cathepsin h (*Ctsh*), Tenascin C (*Tnc*), Lumican (*Lum*), Argininosuccinate synthase 1 (*Ass1*).

The pathway analysis of the top DEGs highlighted anti-microbial (anti-bacterial, anti-fungi, anti-virus) pathways in Adipoq-lineage positive cells in wounds; and highlighted fibrosis, wound healing, and ECM deposition pathways in En1-lineage positive wound fibroblasts (Supplementary Fig. 1j). The genes with high expression level in each pathway were plotted as a heat map. Those anti-microbial genes were drastically upregulated in day 1 explants and the expression returned to low levels in day 4 explants (Fig. 1k).

In conclusion, our data provide tantalizing evidence that fibroblasts and adipocytes refrain from cross-converting between lineages or de-differentiating in whole-skin explant models, and remain genetically bound to their original fibroblast or adipocyte identity.

### Terminally differentiated adipocytes mobilize and reposition during injury

To study the behaviour of mature, terminally differentiated adipocytes and fibroblasts in response to injury, we employed whole-skin explants, allowing visualization and tracking of resident cells, in unprecedented detail over five days in culture, thereby overcoming restrictions associated with short imaging durations in vivo^36^. Ex vivo models, such as this, allow delineation of the phenotypic and behavioural responses of stromal cells after tissue damage. Briefly, whole-skin explants were isolated from the back-skin of both lineage reporter mouse strains and grown in a specialized incubation chamber that maintains constant O_2_/CO_2_, humidity and temperature. We also modified the incubation chamber with specialized silicone rings, to allow multi-photon microscopy objectives to record GFP^+^ adipocytes and fibroblasts within the explants, in high resolution, without compromising environmental conditions. Using this system, explants remained viable and underwent contraction, scar formation and re-epithelialization^36^.

**Fig. 2:**
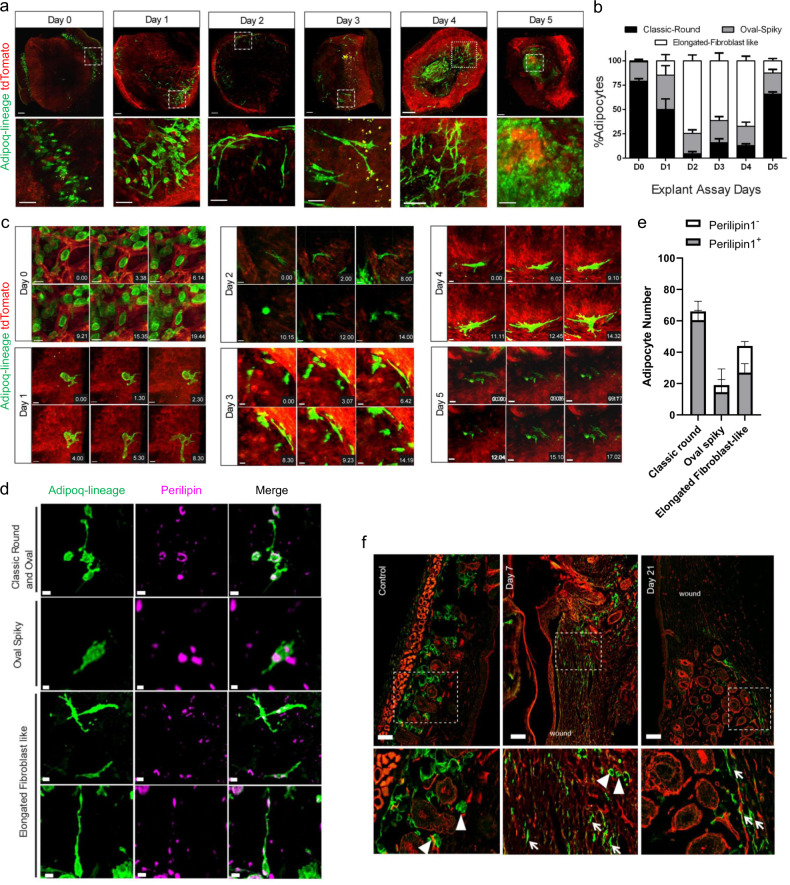
Spatio-temporal characterization of migratory adipocytes. **a** Morphological changes of adiponectin-lineage positive cells (GFP) in skin explants from Adipoq^Cre^;R26^mTmG^ neonates in culture from Day 0 to Day 5 at low (upper panel, scale bar 200 µm) and high (lower panel, scale bar 50 µm) magnification. **b** Quantification of adipocyte morphologies throughout 5-day explant assay, n = 3 explants per timepoint, mean ± SD. **c** Morphology dynamics of adipocytes. Snapshots of single representative cells from Day 0 to Day 5 showing transition from characteristic mature round to migratory states of adipocytes. Time format-hour.min. Scale bar: 20 microns. **d** Three migratory morphologies of adipocytes are positive for Perilipin1 by immuno-labelling. Scale bar: Classic round 20 µm, Oval spliky:10 µm and Elongated fibroblast-like morphologies: upper panel 20 µm, lower panel 30 µm. This experiment was repeated three times independently with similar results. **e** Quantification of Perilipin1-positive and -negative cells in explants, n = 3 biological repeats, mean ± SD. **f** Adipocytes superficially resemble fibroblasts after wounding in live mice: Control back skin of Adipoq^Cre^;R26^mTmG^ mice, GFP^+^ adipocytes are round, located around hair follicles. Following the wound healing experiment at day 7, Adipoq-lineage (GFP) cells seen at the wound periphery have a fibroblast-like elongated morphology. At day 21 after injury, activated adipocytes still have a fibroblastic shape as the skin tissue is undergoing remodelling. Arrowheads indicate round adipocytes, and arrows indicate elongated, activated fibroblast-like cells. Scale bars: 50 µm. This experiment was repeated three times independently with similar results.

With this set-up we obtained time-lapse videos, directly after explant culture, of clusters of mature and round adipocyte bundles, in both the subcutaneous fascia and in the papillary dermis where the bundles wrapped around individual hair follicles (Fig. 2a, Supplementary Movie 1). Adipocyte-lineage cells remained stationary initially, with the first signs of morphologic change visible at around twenty minutes, including the appearance of small filaments or protrusions extending from the cell membrane (Fig. 2b, c). At twenty-four hours, explants had adipocyte-lineage clusters that continued the morphologic transformation, with multiple round adipocytes acquiring oval and spindle-shaped morphologies (n = 110 cells, n = 3 explants). Two days after wounding, 36% of all adipocytes in papillary and fascia deposits gained filopodial extensions (Fig. 2b) that elongated to >100 µm (length of individual cell). Supplementary Movies 2 and 3 illustrate two stages of this morphologic change. Supplementary Movie 2 (and Fig. 2c) shows oval adipocyte-lineage cells extending small membrane filaments with membrane ruffling, indicative of active cytoskeletal reorganization seen in motile cells. Supplementary Movie 3 shows a back-and-forth change of round-to-elongated morphology during movement. Explants on day 2 and day 3 illustrate further filopodia-like extensions, with cells acquiring sleek and slender cell bodies, resembling motile cells. Morphologically transformed adipocyte-lineage cells were interspersed with individual adipocytes adopting a fibroblast-like bipolar morphology, stretching to about 50-200 μm, whereas rounded adipocytes were much smaller, between 10-30 μm. As suggested by our transcriptomics data, adipocytes acquired mobility, moving away from their original fat deposit towards the formative scar region. For example, a single illustrative cell repositioned 380 μm away from its original location (Supplementary Movie 4). Furthermore, elongated adipocytes originated from multiple niches, including fat deposits within the papillary dermis surrounding hair follicles and from within the subcutaneous fascia. On day 4, motile adipocytes, had reached the centre of the scar region but still retained their elongated morphology (Fig. 2a). Supplementary Movie 5 (and Fig. 2c) shows an adipocyte acquiring a bipolar elongated morphology with extending filopodia (11.11 hrs), with a gradually protruding cell body (14.23 hrs).

A succession of snap-shot images of whole-skin explants indicated that adipocyte-lineage cells constantly revert back-and-forth between stationary and migratory states, with accompanying dynamic remodelling of cellular morphology between rounded and elongated/spindle-shapes (Fig.2b, Supplementary Movie 3). Those migrating adipocyte-lineage cells that had relocated at day 5 moved into the centre of the explants where they reverted to their original, lipid-filled, round morphology (Fig. 2a, Supplementary Movie 6, Supplementary Fig. 2). Quantification of morphologies of adipocyte-lineage cells revealed a gradual decrease of rounded cells from day 0 to day 2 and an increase of oval, spindle shaped and elongated morphologies. A reversal of this phenotype was seen back to a strictly rounded morphology between days 3 and 5 (Fig. 2b). All forms of adipocyte-lineage cells including migratory ones retained Plin1 expression (Fig. 2d).

Next, we investigated if mature adipocytes migrated and repositioned in tissues, in the same way as in the ex vivo explant model, in in vivo splinted wounds on the dorsal backs of Adipoq^Cre^; R26^mTmG^ adult mice. Confocal imaging of wounds at day 7 revealed adipocyte-lineage cells clearly acquired fibroblast-like morphologies and translocate from their initial location within dermal white adipose tissue into the wound bed. On day 21, multiple elongated adipocyte-lineage cells aggregated near the scar region, whereas adjacent non-wounded skin adipocytes retained the classical round morphology associated with dermal white adipose tissue (Fig. 2f). These findings confirm that transcriptional changes associated with motility and phenotypic shifts are functionally relevant, with adipocytes transitionally switching morphology and actively migrating, while retaining adipocyte-committed identity.

### Absence of cell fusion events between mature adipocytes and fibroblasts

Having found mature adipocytes switching morphology and migrating both in vitro and in vivo, we wanted to formally exclude all possibility of lineage crossover, therefore we examined the possibility of cell fusion between adipocytes and fibroblasts. Cell fusion includes cytoplasmic intermixing, a phenomenon that can be observed and quantified using red and green lineage-specific fluorescence reporters. Cytoplasmic intermixing would be evidenced as co-labelling of green and red (yellow fluorescence) within migratory adipocytes and fibroblasts. To do this, we analysed and quantified single cells from Adipoq^Cre^;R26^mTmG^ (n = 122) and En1^Cre^;R26^mTmG^ lineages (n = 135) in both tdTomato^+^ and GFP^+^ background at various stages (days) of the explant assay. Lack of co-localization between green and red labels demonstrated that both cell populations, and wound myofibroblasts, shared no history of cell fusion (Supplementary Fig. 3). Collectively, our findings prove that during skin injury adipocytes and fibroblasts remain transcriptionally, behaviourally, and functionally distinct cells, with no inter-conversion or cell fusion between these two stromal lineages.

### Distinct migratory behaviours in adipocytes and fibroblasts

Live imaging data indicated that migratory adipocytes share morphological features with fibroblasts despite there being no cross-lineage plasticity or fusion. To visualize and analyse these distinct motility features, and determine whether adipocytes and fibroblasts share migratory behaviours, we crossed both adipocyte and fibroblast mouse lines with a nuclear mCherry reporter (R26^LSL-H2B^-^mCherry^), allowing tracking of individual cellular nuclei across whole skin. Furthermore, this technique allowed us to compare migratory patterns and features such as velocity, distance travelled, directionality, and collectivity between migrating fibroblasts and adipocytes. Back-skin explants were harvested from Adipoq^Cre^;R26^LSL-H2B-mCherry^ and En1^Cre^;R26^LSL-H2B-mCherry^ double-transgenic mice and cell migration videos recorded in 3D.

**Fig. 3:**
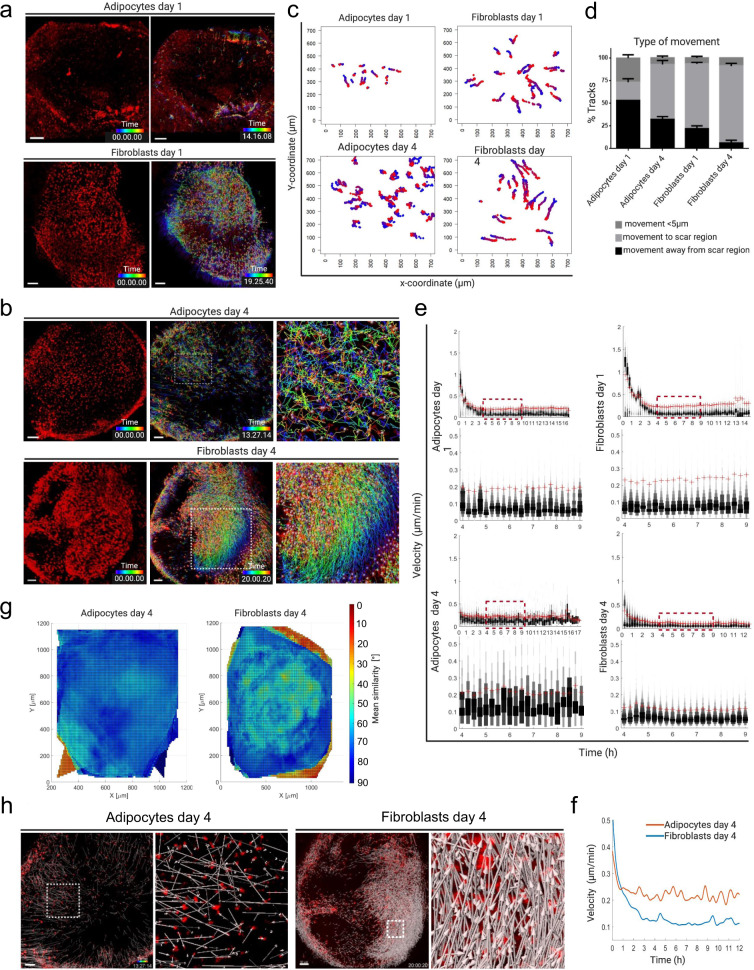
Distinct adipocyte and fibroblast migrations. 3D whole mount time-lapse imaging snapshots of single-cell tracks skin explants generated from Adipoq^cre^ or En1^Cre^ crossed to R26^LSL-H2B-mCherry^ reporter mice. **a** Snapshots of adipocyte- and fibroblast-migration tracks on day 1. **b** Adipocyte and fibroblast tracks on day 4, generated by automated cell tracking using Imaris version 9.2. (Bitplane). **c** Manual tracks of adipocytes and fibroblasts in the scar region of explants at day 1 and day 4; the plot shows the difference in migration distance and type of movement in the scar region of both adipocytes and fibroblasts. N = 2 videos per time point. Scar regions were cropped (700 µm X 700 µm) from whole explant and cells manually tracked. Blue indicates starting time and red is the end-point. **d** 3 main types of movement quantified using manually annotated single cell tracks present in c, n = 3 biological repeats, mean ± SD. **e** Velocity of migratory adipocytes and fibroblasts is calculated using time-lapse videos and automated single cell tracks. Velocity variation and amplitude difference from time point 4 −9 hours across all samples are shown in higher magnification (lower panel). The red crosses (+) indicate the mean velocities of the indicated time points. **f** Spline graph of day 4 showing differences of mean velocity between adipocytes and fibroblasts. **g** Neighbour similarity analysis of day 4 explants using automated single-cell tracks generated from 3D time lapse videos. The colour bar represents the movement angles 0° (red, coordinated movement) to 90° (blue, random movement). Fibroblast migrations are coordinated and collective, whereas adipocyte migrations are random and individual. **h** Directed and non-directed movement of fibroblasts and adipocytes respectively at day 4. Scale bars:100 µm.

Adipocyte-lineage cells initiate their migration from their fat deposits within hours after culture (Fig. 3a, Supplementary Movie 7). On day 1, manual tracking of cells in the scar region revealed that 53% of all evaluated adipocytes move away from their original position, yet only 20% directionally moved towards the formative scar region (defined by a minimal movement of 5 μm) at day 1 (Fig. 3b, c). Fibroblasts, by comparison, rapidly relocating in explants. At day 1 after culture, around 70% of fibroblasts had moved towards the formative scar region, with only 22% of the fibrogenic cell lineage moving in other directions (Fig. 3a, b and Supplementary Movie 8).

On day 4, both adipocytes and fibroblasts remained motile but with widely different patterns of migration. Migration of adipocyte-lineage cells was stochastic at a population level, with individual cells generating migration tracks that were non-coordinated (Fig. 3d, and Supplementary Movie 9). In contrast, groups of hundreds of fibroblasts migrated collectively, in a uniform and coordinated manner, towards the formative scar region (Fig. 3d, Supplementary Movie 10). This indicates that most of the fibrogenic lineage cells respond by directional cell migrating into wounds, whereas adipocyte-lineage cells appear to respond and migrate less purposefully after culture.

Migration dynamics were further quantified using automated cell tracking in the whole explant. The average velocity of adipocyte migration differed significantly from fibroblasts, particularly at day 4. The velocities for individual adipocyte-lineage cells had greater variability throughout all time points, whereas fibroblast velocities remained consistent (Fig. 3e, f). Furthermore, movement similarity analysis confirmed that adipocyte-lineage cells move randomly, in contrast to fibroblasts (Fig. 3g). This suggests that fibroblasts act collectively, whereas adipocytes behave individually. Indeed, directional analysis of adipocyte-lineage cells and fibroblasts further confirmed the collective migration path of fibroblasts, in contrast to adipocyte-lineage cells (Fig. 3h). These findings indicate that migratory adipocytes and fibroblasts respond in a vastly different manner in terms of velocity, directionality, and collectivity. It further indicates that collective cell migration is a defining characteristic of migratory fibroblasts, but not adipocytes.

### Adipocytes do not contribute to scar formation

Accretion of extracellular connective tissue matrix is another defining characteristic of fibrogenic cells^37^. We previously demonstrated that the fibroblasts that go on to have the ability to form scars (by depositing ECM and accumulating dense fibrous tissue) express the *Engrailed* gene temporarily during embryogenesis^25^. We thus compared, the scar-competent ‘Engrailed Past Fibroblasts’ (EPFs) with adipocytes, for their ability to express and secrete ECM proteins in skin explants; mature adipocytes were coloured green with GFP from Adipoq^Cre^;R26^mTmG^ mice, and the scar-forming fibroblasts, EPFs, were coloured red with tdTomato.

**Fig. 4:**
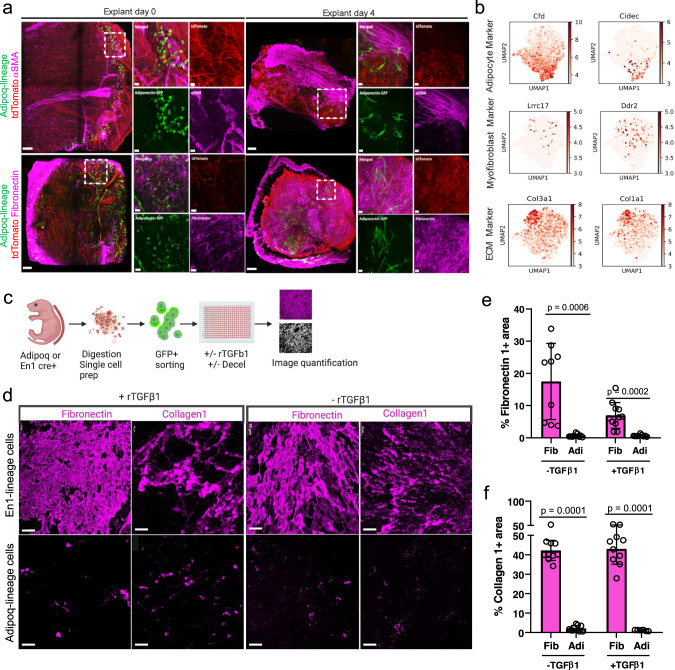
Adipocytes are non-fibrogenic in ex vivo models. **a** Immunostainings of Adipoq^Cre^;R26^mTmG^ explants at day 0 and day 4. Adiponectin-lineage cells in green, fibroblasts in red, and marker gene αSMA (top) and Fibronectin 1 (bottom) expression in magenta. Merged channel image of the whole explant (left), magnified area of individual channels (right). Scale bars: 100 µm in low magnification images, 20 µm in high magnification images. This experiment was repeated three times independently with similar results. **b** Feature plots generated from combined analysis of mcSCRBseq showing adipocyte, myofibroblast, and extracellular matrix-specific enrichment in cell type-specific clusters. **c**-**f** Adipocyte-lineage cells deposit marginal matrix proteins than fibroblasts under scarring conditions. **c** Schematic of in vitro matrix deposition assay and quantification using Image J. **d** FACS-sorted adipocytes, and fibroblasts were cultured in vitro, with and without rTGFβ1 stimulation for 72hrs, followed by decellularization and immunolabelling of deposited matrix Collagen 1 and Fibronectin 1. Scale bars: 50 µm. **e**,**f** Quantification of percent fluorescence of deposited matrix showing higher percentage of deposited matrix when compared to adipocytes, n = 3 biological replicates and 4 images of each replicate, Mean ± SD, Two-way ANOVA with 95% CI.

Expression of the myofibroblast marker αSMA^38,39^ was assessed by immunohistochemical localization at day 0. Here expression patterns correlated with a perivascular niche environment, in the absence of established scar tissue in explants. At day 4 parallel fibrils of αSMA were evident within the scar region, and the fibroblasts started to retract from the developing fibrotic tissue. At this time the adipocyte-lineage cells were distant from the scar developing region, suggesting a less active role in deposition. Furthermore, in day 4 explants the ECM protein Fibronectin was deposited by GFP^-^ tdTomato^+^ fibroblasts and not by GFP^+^ adipocyte-lineage cells (Fig. 4a). Migratory adipocytes did not express fibroblast proteins Fibroblast Specific Protein 1 (FSP1), Transcription Factor 21 (TCF21), or Col3a1 (Supplementary Fig. 4a-e). Myofibroblast and fibrogenic markers, such as *Ddr2*, *Lrrc17*^33,34^, and ECM markers *Col1a1* and *Col3a1*, were upregulated across all fibroblast subsets, but not within adipocytes (Fig. 4b).

To evaluate directly whether adipocyte-lineage cells produce and deposit ECM proteins, we performed 3D immuno-labelling of deposited ECM proteins (Fig. 4c). Adipocyte-lineage cells showed minimal contribution of Fibronectin or Type-I Collagen, irrespective of their morphology or motility status (Fig. 4d). As a control, baseline matrix production was compared to that induced by recombinant transforming growth factor beta 1 (TGFβ1) stimulation, a known pro-fibrotic cytokine upregulated during the early stages of the wound healing response^40^. Fibroblasts deposited significant amounts of Fibronectin and Type-I Collagen, whereas adipocyte showed minimal contribution in ECM production (Fig. 4d-f). Stimulation with TGFβ1 significantly increased Fibronectin and Type-I Collagen in En1-lineage fibroblasts, but not in adipocyte-lineage cells (Fig. 4d-f). Interestingly, TGFβ1 altered cellular contour to the same fibroblast-like morphology seen in migratory adipocyte-lineage cells in vivo (Supplementary Fig. 4f). Despite their morphologic conversion then, we have seen that adipocyte-lineage cells are not fibrogenic, and they do not contribute to the ECM, which is exclusively deposited by fibroblasts.

**Fig. 5:**
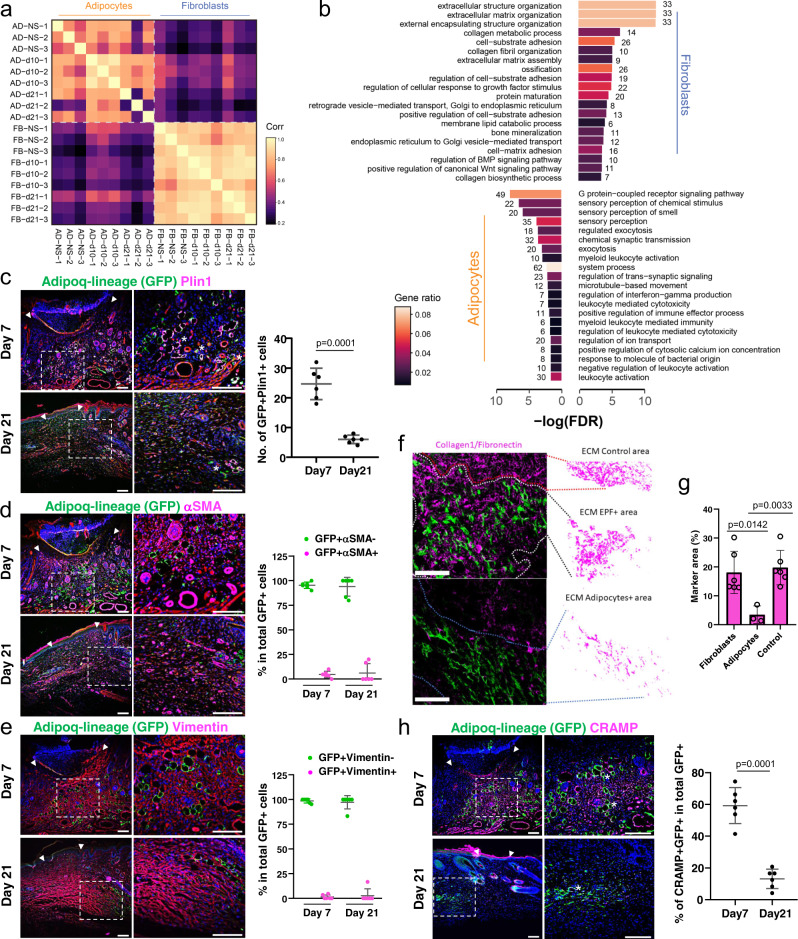
Adipocytes are non-fibrogenic in wounds. mRNA-seq was performed with FACS sorted adipocytes and fibroblasts from day 7 and 21 wounds and adjacent skin of Adipoq^Cre^;R26^mTmG^ and En1^Cre^;R26^mTmG^ mice, respectively. Each cell type at each time point includes three independent biological replicates. **a**. Pearson correlation analysis of all 18 samples. Colour in each cells represented Pearson correlation coefficients for every pairwise comparison. **b** GO term enrichment based on DEGs of adipocytes and fibroblasts from day 10 wounds. Filled colour represented number of genes enriched relative to all DEGs. Cryosections of day 7 and day 21 wounds from Adipoq^Cre^;R26^mTmG^ mice were subjected for immunofluorescence staining. **c** Representative images and quantification of Perilipin (magenta) in GFP positive cells. Data are numbers of GFP^+^Perilipin^+^ cells per high magnification field, n = 6 independent samples, mean ± SD, unpaired two-tailed t-test. **d** Representative images and quantification of αSMA (magenta) in GFP positive cells. The migratory elongated and rounded adipocytes are negative for αSMA. At day 7 there is widespread αSMA staining in the centre of the wound, whereas only physiological αSMA is found in the hair follicle dermal sheath at day 21. Data are percentage of αSMA+GFP+ cells and αSMA-GFP+ cells in total GFP+ cells, n = 6 independent samples, mean ± SD. **e** Representative images and quantification of vimentin (magenta) in GFP positive cells. Data are percentage of Vimentin^+^GFP^+^ cells and Vimentin^-^GFP^+^ cells in total GFP^+^ cells, n = 6 independent samples, mean ± SD. **f** Transplantation of FACS-sorted adipocytes or fibroblasts from P1 new born mice into adult Rag2^-/-^ immunodeficient mouse back skin into an excisional wound model. Immunolabelling with anti-Collagen1 or anti-Fibronectin 1 and quantification of associated extracellular matrix in the transplanted regions. **g** Quantification of adipocyte- and fibroblast-associated ECM in the transplanted regions. n = 3 independent adipocyte samples, n = 6 independent fibroblast or control samples, mean ± SD, unpaired two-tailed t-test. **h** Representative images and quantification of cathelicidin-related antimicrobial peptide (CRAMP) in GFP positive cells. Data are percentage of CRAMP^+^GFP^+^ cells in total GFP^+^ cells, n = 6 independent samples, mean ± SD, unpaired two-tailed t-test. Arrow heads indicate the wound borders, the stars indicate the examples of double positive staining. Scale bars: c-f, h = 100 µm.

We then went on to investigate mature adipocytes in wounds in vivo. Full-thickness excisional wounds were generated on the backskin of adult Adipoq^Cre^;R26^mTmG^ and En1^Cre^;R26^mTmG^ mice, in which the Adipoq-lineage adipocytes and En1-lineage fibroblasts express GFP, respectively. At day 10 and day 21, wounds were harvested and GFP^+^ cells were sorted by FACS. The transcriptomic profiles of the sorted wound adipocyte-lineage cells and fibroblasts were analysed by mRNA-sequencing, with three independent biological repeats for each cell type at each time point. Pearson correlation analysis revealed that adipocytes from day 10 and day 21 wounds were clustered together with adipocytes from adjacent uninjured skin; whereas fibroblasts from day 10, day 21 wounds and healthy skin were clustered together. The transcriptome of the adipocyte cluster was clearly distinct from the fibroblast cluster across all wound time points (Fig. 5a). Gene ontology (GO) enrichment of differentially expressed genes (DEG) in adipocytes and fibroblasts in day 10 wounds indicated that wound fibroblasts and adipocytes performed distinct biological processes. Wound fibroblasts were enriched for cell-matrix adhesion, matrix deposition and regulation. By contrast, wound adipocytes in day 10 wounds were enriched for sensory perception, immune regulation, and anti-microbial responses (Fig. 5b). Comparison across time points showed that the transcriptome of adipocytes from day 21 wounds was closer to adipocytes from the adjacent healthy skin; whereas adipocytes from day 10 wounds upregulated gene features consistent with a migratory mesenchymal cell, which are also highly expressed in fibroblast groups; however, the expression levels of those genes were much lower than En1-lineage fibroblasts (Supplementary Fig. 5a). Still, DEG analysis within adipocytes at different time points showed they retained biological processes of anti-microbial responses, inflammation, and immune regulation (Supplementary Fig. 5b).

Immunofluorescence staining of wound sections showed that the migratory adipocyte-lineage cells were at higher number in day 7 wounds compared to day 21 scars (Fig. 5c). Those migratory adipocyte-lineage cells were negative for fibroblastic markers αSMA (Fig. 5d) and vimentin (Fig. 5e), and proliferation marker Ki67 (Supplementary Fig. 5c), and therefore separated from fibrogenic cells. To evaluate the relevance of these observations further in vivo, we transplanted 2.5×10^5^ individual cells of each lineage into full-thickness dorsal wounds of immuno-deficient Rag2^-/-^ mice. Wounds were harvested 7 days post-transplantation. ECM within the transplanted regions and adjacent non-transplanted areas were quantified as merged immunofluorescence intensities of type-I collagen and fibronectin (Fig. 5f). Transplanted fibrogenic lineage cells generated ectopic scar ECM architectures, whereas transplanted purified adipocyte-lineage cells had negligible effects on scar formation and connective tissue architecture (Fig. 5f, g).

Gene ontology analyses of the adipocyte lineage from both skin explants (Fig. 1j, k) and from in vivo wounds (Fig. 5a, b; Supplementary Fig. 5a, b) indicated they retained anti-microbial responses, immune regulation, and sensory perception, while acquiring a mesenchymal migratory phenotype. To explore the physiological role of adipocytes in wounds, we performed immunostaining of mouse cathelicidin-related antimicrobial peptide (CRAMP), an ortholog of human cathelicidin (the active form is known as LL-37), on day 7 wound section from Adipoq^Cre^;R26^mTmG^ mice. 60% of migratory adipocytes in day 7 wounds showed clear expression of CRAMP, while the expression markedly reduced in adipocytes in day 21 wounds (Fig. 5h). This data confirms that migratory adipocytes retain anti-microbial peptide functions in wounds.

## Discussion

In this paper we demonstrate irreversible cell fate of mature adipocytes during skin injury, despite their remarkable morphological changes and migratory behaviours. Wound adipocytes do not convert their cellular identity into fibrogenic cells, as had been previously assumed^9,10^.

Morphological modulation of wound adipocytes in injured tissues were observed, but there was no evidence for adipogenic origins of fibrosing cells during injury or evidence of conversion or cell fusion from adipocytes to fibroblasts by assessment of transcriptomic, behavioural, or functional criteria.

Our findings that differentiated adipocytes remain committed to their lineages during the healing process, has direct implications to our understanding of homoeostasis and repair of stromal tissues; impacting both basic and clinical paradigms for wound repair, regeneration, and various cell transplantation strategies for regenerative medicine. Clinically, today’s standard of care for skin grafts and scaffold constructs provide closure of a wound area, but leave patients with disfiguring and debilitating scars. Current pre-clinical and clinical studies employ tissue equivalents composed of epidermal and stromal fibroblast populations embedded in various matrices. The incorporation of a dermal component into skin equivalents provides mechanical stability and supports a functional repair tissue by prevention of stricture and scar formation. Clinically approved grafts, such as Apligraf®, have already received Food and Drug Administration (FDA) approval for usage in venous leg ulcers and diabetic foot ulcers. Clear delineation of stromal subsets, and understanding of their tissue specific roles and responsiveness to the environmental milieu, is crucial in the development of novel skin substitutes that enable superior ‘scarless’ regenerative outcomes in the treatment of severe skin defects, burns, accidents, congenital diseases, tumours, or chronic wounds.

Fat grafting, or lipofilling, is another clinical example where lineage conversions from adipocytes to fibroblasts have been proposed to enhance tissue repair processes not only by providing tissue volume, but also by reducing scar formation and fibrosis, thereby improving the functionality of the repair tissue^41^. Convincing clinical evidence for the effectiveness of fat grafting remains elusive. Data from our study highlights that transplanted mature adipocytes will not morphos into stromal fibroblasts, contributing to repair tissue; rather this remains the feat of the resident stromal fibroblasts. However, key reparative signals and a supportive milieu provided by the adipocytes may promote the function and response of the injured stroma. This remains an area warranting further investigation and clinical refinement as our knowledge deepens.

Previous studies from Horsley’s group suggested a conversion of wound adipocytes to myofibroblasts by using lineage tracing coupled with scRNA-seq^9,10^. However, the cell numbers of tracked GFP expressing adipocytes in wounds included in the analysis was extremely low, and in the background noise of large numbers of fibroblasts and contaminated leukocytes and endothelial cells^9^. Conversion was reported based on scRNAseq data from 13 wound adipocytes, whereas our analysis was conducted on a much larger population (200 − 2000 wound adipocytes per sample). Nonetheless, even if the conversion happened in such few cells, it remains unclear whether they play physiologic roles in scar formation, where the predominant numbers of myofibroblasts originate from En1^+^ fibroblast lineages. In our mRNA-seq analysis of in vivo wound fibroblasts and adipocytes, we used bulk RNA-seq with FACS enriched En1-lineage positive fibroblasts and Adipoq-lineage positive adipocytes, ranging from 200 to 2000 cells per sample, which largely increased the cell numbers of input adipocytes, and therefore remarkably reduced the noise. There are a couple of limitations to this study. The scRNA-seq data is derived from skin explants, which lack immune cells, blood supply and innervation, all of which could influence the transcriptomics data and therefore we have confirmed functional separation of fibroblasts and adipocytes in vivo in wounded animals. Another limitation relates to the preparation procedure of purifying adipocytes that is considered to be technically challenging and therefore relatively low numbers of adipocytes are analysed, as compared to fibroblasts. Nevertheless, we believe the sequencing analyses presented in this study are closer to the genuine physiologic roles of wound adipocytes.

The scRNA-seq analysis of adipocyte in the whole-skin explant model, and the mRNA-seq data of adipocytes in excisional wounds have suggested that the migratory mature adipocytes are involved in anti-microbial responses, immune regulation, and sensory perception. The adipocytes in day 7 wounds express high level of cathelicidin-related antimicrobial peptide, confirming their anti-microbial functions in vivo. In line with these findings, skin adipocytes have been shown to execute anti-microbial functions during injury repair or skin infection, by releasing anti-microbial peptide cathelicidin^42,43^. Such behaviour is believed to be regulated by toll-like receptors and retinoic acid^44^. Furthermore, the antimicrobial function of wound adipocytes is evolutionary conserved. Fat body cells, the equivalent of vertebrate adipocytes in *Drosophila* have been demonstrated by live imaging that they participate in wound healing, by actively migrating to wounds and physically sealing wounds. The fat body cells also help to fight against the infection by locally releasing antimicrobial peptides such as Attacin^45^. The other predicted physiological functions of wound adipocytes such as immune regulation and sensory perception require further investigation.

This study delineates multi-modality in lineage-restricted adipocytes in their response to tissue injury. Understanding the limitations of lineage interplay between stromal cells provides key knowledge to tissue repair responses central to multiple organ systems, as well as supporting translational knowledge for the development of novel tissue engineering and stromal cell-based therapeutics for impaired healing and fibrotic disorders.

## Methods

### Transgenic & reporter mouse lines

This study complies with all relevant ethical regulations. All animal experiments were reviewed and approved by the Government of Upper Bavaria and registered under the projects under projects ROB-55.2-2532.Vet_02-16-61, ROB-55.2-2532.Vet_02-19-23, and ROB-55.2-2532.Vet_02-21-153, and conducted under strict governmental and international guidelines. C57BL/6 J wild type mice were purchased from Charles River (strain code 632). En1Cre (JAX stock No. 007916), ROSA26LSL-H2B-mCherry (R26^LSL-H2BmCherry^) (JAX stock No. 023139), Rag2^–/–^ mice and ROSA26mTmG (R26^mTmG^) reporter mice were from Stanford University^25^. Adipoq^Cre^ (Jax stock No.028020) mice were from Helmholtz Center. En1^Cre^ or Adipoq^Cre^ transgenic mice were crossed with R26^mTmG^ or R26^LSL-H2BmCherry^ reporter mice for use in the described experiments. Animals were housed at the Helmholtz Center animal facility rooms that were maintained at constant temperature and humidity with a 12-h light cycle. Animals were given food and water ad libitum. No sex selection was performed in this study.

### Mouse genotyping

Cre^+^ animals from double-transgenic reporter mice were classified based on appropriate fluorescence present in the dorsal back of the mice. A fluorescence microscope was used for identification of Cre^+^ neonatal mice. For adult mice, genotyping was performed to detect a 200 base pair Cre band (fragment). Filter tips were used to prevent cross contamination. Genomic DNA was extracted normally from ear clip tissue. Quick Extract (QE) DNA extraction solution (Biozym, 101094) was used. Extracted Genomic DNA solution 1 µl, was added to each PCR reaction mix containing 24 µl. The PCR reaction mixture was prepared using Taq PCR core kit (Qiagen, 201205) containing Coral buffer (1× concentration final), dNTPs (10 mM each), Taq polymerase enzyme (0.625 units per reaction), 0.5 µM of each forward primer (FP) 5′ ATT GCT GTC ACT TGG TCG TGG C-3′ and reverse primer (RP) 5′ GGA AAA TGC TTC TGT CCG TTT GC-3′. PCR cycling temperature was set to 10 min at 94 °C for initial denaturation. Followed by amplification of 30 cycles: 30 s denaturation at 94 °C, 30 s annealing at 56 °C, 30 s elongation at 72 °C. The final elongation at 72 °C was for 8 min, and an infinite temperature of 10 °C was maintained. Negative controls with no template and positive controls were included. The Eppendorf master cycler instrument was used, and samples were analyzed by agarose gel electrophoresis.

### Ex vivo explant culture

The skin-fascia explant assay was performed by following our established protocol^36,46^. Briefly, back-skin was collected from new-born (postnatal day 0–1) two-colour membrane reporter En1^Cre^;R26^mTmG^ or Adipoq^Cre^;R26^mTmG^ reporter or En1^Cre^;R26^LSL-H2B-mCherry^ mice or Adipoq^Cre^;R26^LSL-H2B-mCherry^ nuclear reporter mice, and washed twice with DMEM/F-12 (Thermo Fisher Scientific, 11320074) medium to remove contaminating blood, and then washed once with Hank’s Balanced Salt Solution (HBSS, Thermo Fisher Scientific 14175095). Dorsal back skin was cut out and explants were made using a disposable Ø 2 mm biopsy punch (Stiefel, 270130) down to below the panniculus carnosus muscle, and cultured in 2 ml of DMEM/F-12 medium containing 10% FBS, 1× GlutaMAX (Thermo Fisher Scientific, 35050038), 1× Penicillin/streptomycin (Thermo Fisher Scientific, 15140122), and 1× MEM non-essential amino acids (Thermo Fisher Scientific, 11140035) in 6 well plates, in a humidified 37 °C, 5% CO_2_ incubator. Fresh medium was supplied every other day and the skin tissues were harvested at the indicated time points (day 1 to day 5 after culture), with the fresh tissues serving as day 0 control. Post harvesting on designated days, explants were fixed in 2%, paraformaldehyde (PFA), overnight at 4 °C for whole mount imaging and immuno-labelling, or directly used for live imaging by multiphoton microscopy or mcSCRB-seq experiments.

### Sorting of adipocytes and fibroblasts

Transgenic mouse lines (En1^Cre^;R26^mTmG^ or Adipoq^Cre^;R26^mTmG^) at P0 or P1 stage were used for sorting. Multiple skin explants for each day was pooled to maximize yield. Tissue was minced and digested using 0.5 mg/ml Collagenase A (Sigma Aldrich, 10103586001) and 25 U/ml of DNase 1 (Sigma, 10104159001) for 1 hr at 37 °C with shaking. Cells were washed with complete media and filtered through cell strainers. Centrifugation was carried out for 5 min at 200 g and the cell pellet was resuspended in 1 ml of FACS buffer containing 2% FBS in PBS. Adipocyte and fibroblast sorting were performed based on GFP^+^ fluorescence. En1cre^+^ fibroblasts were incubated with the following antibodies for lineage-negative gating. A total of 1 μg of APC-conjugated anti-mouse CD45 (BioLegend, 103112, 1:200), EpCAM (CD326) (BioLegend, 118214, 1:200), PECAM1(CD31) (BioLegend, 102410, 1:200), Ter119 (BioLegend, 116212, 1:200), Tie2 (CD202b) (BioLegend, 124008, 1:200) and eFluor660-conjugated anti- LYVE1 (eBioscience, 50-0443-82, 1:200) in FACS buffer (2% [v/v] FBS in phosphate buffered saline [PBS]), on ice for 30 min. The antibody-conjugated cell pellet was washed in 5 ml FACS buffer then re-suspended in 1 ml of FACS buffer (PBS + 2% FBS). The cells were sorted using the a FACS Aria III sorter with a 120 μm nozzle. Fibroblasts were sorted based on the following gating scheme: Lin-TomatoRed^-^GFP^+^ from En1^Cre^;R26^mTmG^. Adipocytes were sorted based on the following gating scheme: TomatoRed^-^GFP^+^ from Adipoq^Cre^;R26^mTmG^.

### Cell sorting for mcSCRB-seq

Dorsal back skin was taken from En1^Cre^;R26^mTmG^ or Adipoq^Cre^;R26^mTmG^, postnatal day 0 or day 1 mice and washed three times with HBSS. The tissue was minced and incubated with 5 ml of digestion mix for preparation of single cell suspension. mcSRCB-seq was performed with 1000 adipocytes or fibroblasts sorted from day 1 and day 4 explants. The indexing parameter in Aria III was used and cells were sorted directly into 96-well DNA LoBind plates (Eppendorf). Each well was aliquoted with 5 µl lysis buffer prior to sorting. Lysis buffer consisted of Guanidine Hydrochloride (5 M concentration; Sigma Aldrich), β-Mercaptoethanol (1%, Sigma Aldrich), and Phusion High Fidelity (HF) buffer (1:500 dilution, New England Biolabs: M0531L). Immediately post sorting, plates were spun down, placed on dry ice, and later stored at −80 °C.

### cDNA synthesis step of mcSCRB-seq (pre amplification step)

A full step by step protocol for mcSCRB-seq is deposited in the protocols.io repository^27^. Before the preparation of libraries, SPRI beads were used to clean up each well. Beads were resuspended in 4 µl double distilled water along with a reverse transcription master mix (5 µl) containing Maxima H-RT enzyme (20U, Thermo Fisher), Maxima H buffer 2x (Thermo Fisher), dNTPs at concentration 2 mM each (Thermo Fisher), template switching oligo (from IDT) 4 µM and Poly ethylene gycol (PEG) 8000 15% (Sigma-Aldrich). After addition of 1 µl of 2 µM barcoded oligo-dT primer (E3V6NEXT, Integrated DNA technologies), cDNA was prepared according to the mcSCRBseq protocol. Template-switching and cDNA synthesis was performed at 42 °C for 90 min. Barcoded cDNA was later pooled in 2 ml DNA LoBind tubes followed by clean-up using SPRI bead buffer. Purified cDNA was eluted in 17 µl and residual primers digested with enzyme Exonuclease I (from Thermo Fisher) at 37 °C for 20 min. After heat-inactivation at 80 °C for 10 min, 30 µl PCR master mix containing Terra direct polymerase, 1.25 Units (Clonetech), Terra direct buffer 1.66 X, and SINGV6 primer (IDT) 0.33 µM, was added. PCR was cycling was performed as follows: 98 °C, 3 min for initial denaturation, 19 cycles of 15 seconds at 98 °C, 65 °C for 30 s and at 68 °C for 4 min. Lastly, final elongation was performed at 72 °C for 10 min.

### scRNA-seq library preparation

All samples were purified with SPRI beads (ratio 1: 0.8) after pre-amplification. Final elution was in 10 µL of nuclease-free water (Invitrogen). Later cDNA was quantified using the Quant-iT PicoGreen double stranded DNA Assay Kit (Thermo Fisher). A High Sensitivity DNA chip (Agilent Bioanalyzer), was used to verify size distributions. Samples that passed the quality and quantity control parameter, were used for constructing Nextera XT libraries from 0.8 ng of pre-amplified cDNA. Later, 3’end sequences were enriched with a custom made P5 primer (P5NEXTPT5, IDT), during PCR of the libraries. Following this, Libraries were pooled and selected based on their size using 2% E-Gel Agarose EX Gels (Life Technologies); a size range from 300 bp to 800 bp was cut out and extracted using the MinElute kit (Qiagen). All Procedures were in accordance with the manufacturer’s recommendations.

### Sequencing of scRNA-seq libraries

An Illumina HiSeq 1500 instrument was used to sequence libraries. Paired-end sequencing of libraries was performed on high output flow cells. To generate molecular and cellular barcodes, 16 bases were sequenced with the 1st read, 50 bps sequenced in the 2nd read into the cDNA fragment, and 8 bases were read to obtain the i7 barcode.

### Full-thickness excisional wound model

Adult (8–16 weeks old) Adipoq^Cre^;R26^mTmG^ or En1^cre^;R26^mTmG^ mice were anesthetized with MMF (medetomidine at 500 μg/kg, midazolam at 5 mg/kg and fentanyl at 50 μg/kg body weight). Dorsal back hair was shaved with a hair clipper, and further removed with depilatory cream. Two full-thickness excisional wounds were created with a 5 mm diameter biopsy punch (Stiefel). Mice were supplied with analgesic. Wounds were harvested at days 7, 10, or 21 post-wounding depending on the subsequent analyses.

### Bulk RNA-sequencing of adipocytes and fibroblasts from in vivo wounds

Full-thickness excisional wounds were made on the back of Adipoq^Cre^;R26^mTmG^ mice and En1^Cre^;R26^mTmG^ mice. The digested samples were filtered through 100 µm cell strainers to allow collection of adipocytes with bigger size, and centrifuged at 300 g for 5 min, and floating adipocytes were isolated using a pipette. Adipocytes and fibroblasts were FACS sorted from day 10 and day 21 wounds of respective transgenic lines as GFP^+^ cells. 200 – 2000 FACS sorted cells were lysed and cDNA synthesis and the subsequent expansion with 8 PCR cycles were performed using the Smart-Seq® v4 Ultra® Low Input RNA Kit for Sequencing (Takara #634889), according to manufacturer’s instructions. The amplified cDNA was purified with NucleoMag NGS Clean-up and Size Select beads (Takara 7449705). The library preparation and sequencing were performed by Biomarker Technologies (BMKGene) GmbH (Münster, Germany) with Illumina Novaseq 6000. Standardized RNAseq pipelines written in Nextflow, nf-core/rnaseq were adapted for trimming, alignment to mm10 by STAR and counts calling by salmon as default setting^47^. The raw counts were used for a differential gene expression analysis (DEG) using DEseq2 (version 1.34.0 with R version 4.1.2)^48^. Normalized counts were scaled for Pearson’s correlation matrices and plotting feature genes expression in heat map. Gene ontology (GO) enrichment of DEGs was done using clusterProfiler (version 4.2.2) and viewed by ggplot2 (Version 3.3.6)^49^.

### Primary data processing of RNA-seq data

Processing of all raw FASTQ data was done using zUMIs pipeline along with STAR to obtain expression profiles for barcoded UMI data^28,29^. Mapping was done to the reference genome (mm10/09) concatenated with the ERCC and GFP reference. Gene annotations were obtained from Ensembl (GRCm38/mm10—GRCh38.84).

### Processing of mcSCRB-seq single cell data set

The raw count matrices output by the zUMIs pipeline were analyzed using Scanpy^50^ (v.1.6.0). For barcode filtering, we excluded barcodes with less than 1000 detected genes. We assessed the number of unique molecular identifiers (UMIs) for each sample using violin plots, and retained cells with a number of UMIs below 250000. Genes were only considered if they were expressed in at least 3 cells in the data set.

Normalization was performed based on scran’s approach^51^, in which size factors are calculated and used to scale the counts in each cell. Log transformation was used via Scanpy’s pp.log1p.

Top variable genes were established with scanpy’s pp.highly_variable_genes and flavour set to “cell_ranger”. Highly variable genes were the basis for the principal component analysis (PCA) and neighbourhood graph construction via pp.pca and pp.neighbours (adipocytes: n_pcs = 15, n_neighbors = 5; fibroblasts: (n_pcs = 20, n_neighbors = 10). For clustering the louvain algorithm was employed at resolution 0.5, resulting in 6 clusters within the adipocyte and fibroblast populations, respectively. For both subsets the UMAPs and diffusion maps were generated using Scanpy’s functions tl.umap and tl.diffmap.

As we anticipate a small fraction of contamination with adipocyte cells in the fibroblast labelled population, we cleared away such adipocyte cells. For that we assessed the similarity to adipocytes for each cell in the fibroblast subset with scanpy’s tl.score_genes function, using an adipocyte reference signature (adipocyte cell type from Mouse Cell Atlas, link: http://bis.zju.edu.cn/MCA/gallery.html?tissue=Neonatal-Skin,cluster3). Unsupervised louvain clustering at resolution 1 revealed one cluster with particularly high scoring cells, which was then removed from the analysis.

For the comparison of both lineages, the two refined subsets were re-combined into one object and the list of variable genes set to the union of the list established on the two subsets. The PCA was re-calculated and the neighbourhood graph established for the concatenated object (n_pcs = 15, n_neighbors = 20). Cell type marker genes for the 12 subclusters were established with tl.rank_genes_groups and method = “wilcoxon”.

### Differential Gene Expression across the two lineages

We performed differential expression analysis with diffxpy (v.0.7.4). In a first analysis we compared the differences between adipocyte and fibroblast lineage using the Wald test. To circumvent the problem that certain genes are highly upregulated in only one of the 6 subclusters per lineage, we constrained the model to each subcluster by adding constraint_loc = [subcluster: lineage] to the model. Genes are labelled as differentially expressed if their Benjamini-Hochberg corrected p-value was less than 0.5, have a log2foldchange of greater 1 and are expressed in at least 10% of cells in the relevant lineage. This resulted in a list of 88 genes for adipocytes and 198 genes for fibroblast.

Gene Set Enrichment Analysis was performed using these differentially regulated genes as input for the python package GOATOOLS^52^.

### Whole mount 3D imaging by multi-photon microscopy

Post fixation, explants were thoroughly washed in PBS 3X times, 1 h each, followed by, embedding in NuSieve GTG agarose (2%, Lonza, cat.no. 859081) in a 35 mm dish (Falcon, cat.no. 351008). Imaging was performed under a Leica SP8 Multiphoton microscope (Leica, Germany). Tiles were merged with a LAS X (v4.8, Leica) with smooth overlap blending, and data was finally visualized with Imaris image analysis software (v9.1.0 and v9.2, Bitplane, UK) using contrast and brightness adjustments.

### Live 3D multi-photon microscopy

Live samples were embedded as above, and live imaging was performed using multi-photon microscopy. Imaging medium included DMEM/F-12. Time-lapse imaging was performed over 15 hours under the multi photon microscope. A modified incubation system, with heating and gas control (ibidi 10915 & 11922), was used to guarantee physiologic and stable conditions during imaging. Temperature control was set to 37 °C with 5% CO_2_-supplemented air. 3D data was processed with Imaris 9.1.0 (Bitplane, UK) and ImageJ (1.52i). Contrast and brightness were adjusted for better visibility.

### Manual cell tracking

Manual cell tracking was performed on explants from nuclear reporter lines (En1^Cre^; R26^LSL-H2B-mCherry^ or Adipoq^Cre^;R26^LSL-H2B-mCherry^). ImageJ software with “Manual Tracking” plugin (version 2.1.1) was used. In brief, an area of 700 µm X 700 µm in the scar region of the explant was cropped from 3D-Time lapse datasets. Nuclear spots were identified after subjecting to maximum-intensity projection. Migration of individual cells was tracked over time. Trajectories and individual track information with coordinates were exported as TIFF and excel file respectively. Graphical visualization and analysis of these trajectories were performed using “R”. Colour-ramps were generated for each track as a function of time (Blue; first time point; Red; last time point). Cell movement patterns were quantified based on whether a cell moved away (towards to epidermis) or towards the scar region (center of the explant).

### Automated cell tracking

Automated cell tracking was performed using 3D-Time lapse datasets of whole explants made from nuclear reporter lines using the Imaris software package (v9.2.1, Bitplane, UK). Live videos were generated with a 15 min interval from explant stages of day 1 and day 4. Tracks were generated from 3D data using mCherry fluorescence and an intensity-based spot detection tool. Tracks were visualized in time-coded colour representation, ranging from purple to red. For snapshot images, full tracks representing the last 10 time points were shown for better visibility and to prevent overcrowding. Dragon tail representation was used in live videos to better visualize directed collective and non-directed migration patterns.

### Quantification of cell migration

Employing the tracked cell trajectories, the displacement in 3D for every cell between consecutive time frames was calculated. To compare experiments, mean cell displacements were added up and plotted over time. To analyse the movement similarity of neighbouring cells, the neighbourhood was determined via Delaunay triangulation. Neighbours were defined as cells that are direct neighbours in the resulting neighbourhood graph. Next, the 3D movement vectors for a cell and its neighbours for consecutive time points were calculated. The 3D movement similarity for cell ‘*u*’ and neighbour ‘*v*’ can be assessed by calculating the intermediate angle between the respective movement vectors:

1 $${\alpha }_{u,v}={{{\cos }}}^{-1}\left(\frac{{u}_{x}{v}_{x}+{u}_{y}{v}_{y}+{u}_{z}{v}_{z}}{\sqrt{{u}_{x}^{2}+{u}_{y}^{2}+{u}_{z}^{2}}\sqrt{{v}_{x}^{2}+{v}_{y}^{2}+{v}_{z}^{2}}}\right)$$

Finally, all angles were averaged to produce one movement similarity score. For display reasons the angles were inverted and the movement similarity ranges from 90° (random movement) to 180° (coordinated movement).

### Whole mount and conventional immunostaining

Whole-mount samples were pre-incubated in Dulbecco’s Phosphate-Buffered Saline (DPBS, Thermo Fisher Scientific 14190169) containing 0.2% gelatin (Sigma G1393), 0.5% Triton-X100 (Sigma X100) and 0.01% Thimerosal (Sigma T8784) (PBS-GT), for 24 h at room temperature. They were then incubated with primary antibodies. Primary antibody incubation was carried out at room temperature for 72 h, followed by washing with PBSGT, 3× times, 1 h each. Fluorophore-conjugated secondary antibodies were purchased from Thermo Fisher Scientific. The samples were incubated, rotating, with the labelled antibodies in PBSGT (1:1000) for 24 h at room temperature. Samples were then washed with PBSGT 3× times, 1 h each and stored in fresh PBSGT at 4 °C in the dark until imaging. The following primary antibodies were used: Collagen I (1:200 Rockland, cat.no. 600-401-103-0.1), Collagen III (1:200, Abcam ab7778), Fibronectin 1 (1:250, Abcam ab23750), a-SMA (1:200, Abcam ab5694), Perilipin1 (1:200, Abcam, ab3526), CRAMP (1:200, Novus Biologicals, NB100-98689), FSP1 (1:200, Abcam, ab58597), PDGFRα (1:200, R&D Systems AF1062), Ki67 (1:200, Abcam, ab15580), TCF21 (1:250, Abcam, ab32981), Vimentin (1:200, Abcam, ab8978). AlexaFluor488-, AlexaFluor568-, or AlexaFluor647-conjugated secondary antibodies against suitable species were used for fluorescence labelling, for example: AF488-conjugated goat anti-rabbit IgG (Life Technologies, A11008, 1:500), AF568-conjugated donkey anti-rabbit IgG (Life Technologies, A10042, 1:500), AF647-conjugated donkey anti-rabbit IgG (Life Technologies, A31573, 1:500). The images were taken with a Thunder Imaging Systems (Leica) acquired by Leica Application Suite v4.8, or an AxioImager (Carl Zeiss) acquired by Zen v3.0 blue edition.

### Lipid tox staining

All procedures followed were in accordance with manufacturer’s instructions. HCS Lipid TOX Deep Red (Thermo Scientific, H34477) was used at 1:200 dilution on PFA fixed whole mount explants and incubated at 37 °C for 2 h. Whole mount imaging used a Multiphoton microscope.

### Extracellular matrix deposition assay

In vitro culture of FACS-sorted cells were obtained from transgenic mouse lines (En1^Cre^; R26^mTmG^ or Adipoq^Cre^;R26^mTmG^) at the P0 or P1 stage. Explants from Cre+ mice were collected on day 1 and day 4. Tissue was minced and digested using 0.5 mg/ml Collagenase A and 25 μg/ml of DNase 1 for 1 h at 37 °C with shaking. Cells were washed with complete media and filtered through cell strainers. En1^cre+^ samples were incubated with the following antibodies for lineage-negative gating: APC- anti-mCD31, mCD45, EpCam (CD326), PECAM-1, mTie2(CD202b), mTer119, anti-mLyve-1 on ice for 30 min. The antibody-conjugated cell pellet was then washed with, 5 ml FACS buffer and re-suspended in 1 ml of FACS buffer (PBS + 2% FBS). The cells were sorted by a FACS Aria III sorter with a 120 μm nozzle. EPFs were sorted based on Lin^-^TomatoRed^-^GFP^+^ fluorescence. For Adipoq^Cre^ positive samples, digestion procedures were similar to those mentioned above but excluded lineage markers. En1+ fibroblasts and Adipocytes were later plated in 384 well glass bottom plates coated with 1% Porcine Gelatin. 3000 cells were plated per well and cultured in complete medium with or without 5 ng/ml recombinant TGFβ1 (rTGFβ1). Adipocytes were cultured in complete medium with or without 5 ng/ml rTGFβ1 and 5 ng/ml bFGF (basal FGF). Both cultures were maintained in 37 °C incubator for 72 h and media was replenished once.

### Decellularization and extracellular matrix immunostaining

Adipocytes and fibroblasts in 384-well plates were cultured up to 3 days. Confluent fibroblasts or adipocytes were de-cellularized using an established protocol^53,54^. Briefly, confluent culture dishes were incubated at 4 °C for 45 min with very gentle stirring, sequentially, first in DDW (double distilled water) with Triton X 100 (0.1%) at 4 °C, then in freshly prepared 2% sodium deoxycholate. This was followed by two 30-min incubations at room temperature, first in DDW containing 1 M NaCl and second in DDW containing pancreatic DNase (30 μg/ml, source porcine), MgSO_4_ (1.3 mM) and CaCl_2_ (2 mM). Plates were later rinsed very gently with distilled water, and 4% PFA was used to fix the deposited extracellular matrix for 15 min at RT. After washing twice gently with PBS, permeabilization was performed in PBS containing BSA (1%) and Triton X (0.1%). Blocking was carried out for 1 h at RT in PBS containing 1% BSA and 10% serum of the species in which secondary antibody was raised. Next followed a primary antibody incubation at 4 °C overnight, gentle rinsing with PBS and later secondary antibody was applied for 1 h at RT. Finally, nuclei were stained with DAPI for 10 min at RT. Plates were washed 3× times with 1× PBS and stored in PBS, and images were taken with a confocal microscope (LSM 710, Zeiss). Percent fluorescence was measure after converting the images to binary format in Image J and calculating the area of the fluorescence signal.

### Cell transplantation

FACS sorted cells were first thoroughly and gently washed with PBS. Cells were later resuspended in PBS at 5 × 10^5^ cells per ml. An equal volume of ice-cold Matrigel was mixed with the sell suspension (Phenol red free, Corning, 356231). Cell suspensions in Matrigel were made to a final concentration of 2.5 × 10^5^ cells per ml and stored in an ice bucket until intradermal injections. Rag2^−/−^ mice, at age 10–12 weeks, received adipocyte lineage and fibroblast lineage cell—Matrigel transplants. A total of 5 mm diameter excisional wounds (full-thickness, 2 wounds) were made on the dorsal back skin of mice, as described above. Two 50 μl injections of adipocyte lineage and fibroblast lineage cell-Matrigel suspensions were made near the wound site. Two similar intradermal injections of 50 μl PBS alone were made for control wounds. Cell-Matrigel suspensions were left for 10 min, followed by placing silicone splints around the wound. Gentle press was applied around the wound as mentioned above in the splinted wound method. Whole skin including scar tissue was harvested 7 days post wounding. Lastly, harvested tissue was fixed at 4 °C, overnight using 2% PFA. After 3 PBS washes, the tissues were prepared for histological analysis.

### Statistics and reproducibility

GraphPad Prism 8 was used for all statistical analyses except for sequencing data. Unless otherwise indicated, mean ± SD values are reported in the graphs. The exact statistical analyses used to quantify data, the exact values of *n*, and the exact *p* values are stated in the respective figure legends. For simplicity, *p* values below 0.0001 were stated as equal to 0.0001. All experiments were performed at least three times independently with similar results.

No statistical method was used to predetermine sample size. Required experimental sample sizes were estimated based on previous established protocols in the field. The sample sizes were adequate as the differences between experimental groups were reproducible. No data were excluded from the analyses. The experiments were not randomized. The Investigators were not blinded to allocation during experiments and outcome assessment.

### Reporting summary

Further information on research design is available in the Nature Portfolio Reporting Summary linked to this article.

## Supplementary information

Supplementary Information

Description of Additional Supplementary Files

Supplementary Movie 1

Supplementary Movie 2

Supplementary Movie 3

Supplementary Movie 4

Supplementary Movie 5

Supplementary Movie 6

Supplementary Movie 7

Supplementary Movie 8

Supplementary Movie 9

Supplementary Movie 10

Supplementary Data 1

Supplementary Data 2

Reporting Summary

## Data Availability

The generated scRNA-seq data has been deposited in the Gene Expression Omnibus under the accession number GSE175650. The generated mRNA-seq data has been deposited in the Gene Expression Omnibus under the accession number GSE215912. The raw sequence data has been deposited in the Sequence Read Archive (SRA) with the BioProject ID PRJNA889718. Source data for Figs. 2, 4, 5 has been provided in the Source Data file. Source data are provided with this paper.

## Source data

Source Data

## Code availability

scRNA-seq data analysis was performed with scanpy (v1.6.0) and diffxpy (v0.7.4). Gene set enrichment analysis was performed with goatools (v0.18). Custom R and Python code to reproduce the analyses and figures based on scRNA-seq data generated in this manuscript has been deposited in GitHub [https://github.com/schillerlab/2021_Stromal_Fate_Restriction].
