# Supplementary material for: Wound infiltrating adipocytes are not myofibroblasts
Source: Nat Commun. 2023 May 25;14:3020. doi: 10.1038/s41467-023-38591-6 (PMC10213017; doi:10.1038/s41467-023-38591-6)
Supplement: Supplementary file 1 — Supplementary Information [file 41467_2023_38591_MOESM1_ESM.pdf]

# Wound infiltrating adipocytes are not myofibroblasts

Shruthi Kalgudde Gopal[1,2,#], Ruoxuan Dai[1,#], Ania Maria Stefanska[1], Meshal Ansari[2,3], Jiakuan Zhao[1], Pushkar Ramesh[1], Johannes W. Bagnoli[4], Donovan Correa-Gallegos[1], Yue Lin[1], Simon Christ[1], Ilias Angelidis[2], Valerio Lupperger[3], Carsten Marr[3], Lindsay C. Davies[5], Wolfgang Enard[4], Hans-Günther Machens[6], Herbert B. Schiller[2,*], Dongsheng Jiang[1,*], and Yuval Rinkevich[1,*]

## SUPPLEMENTARY INFORMATION

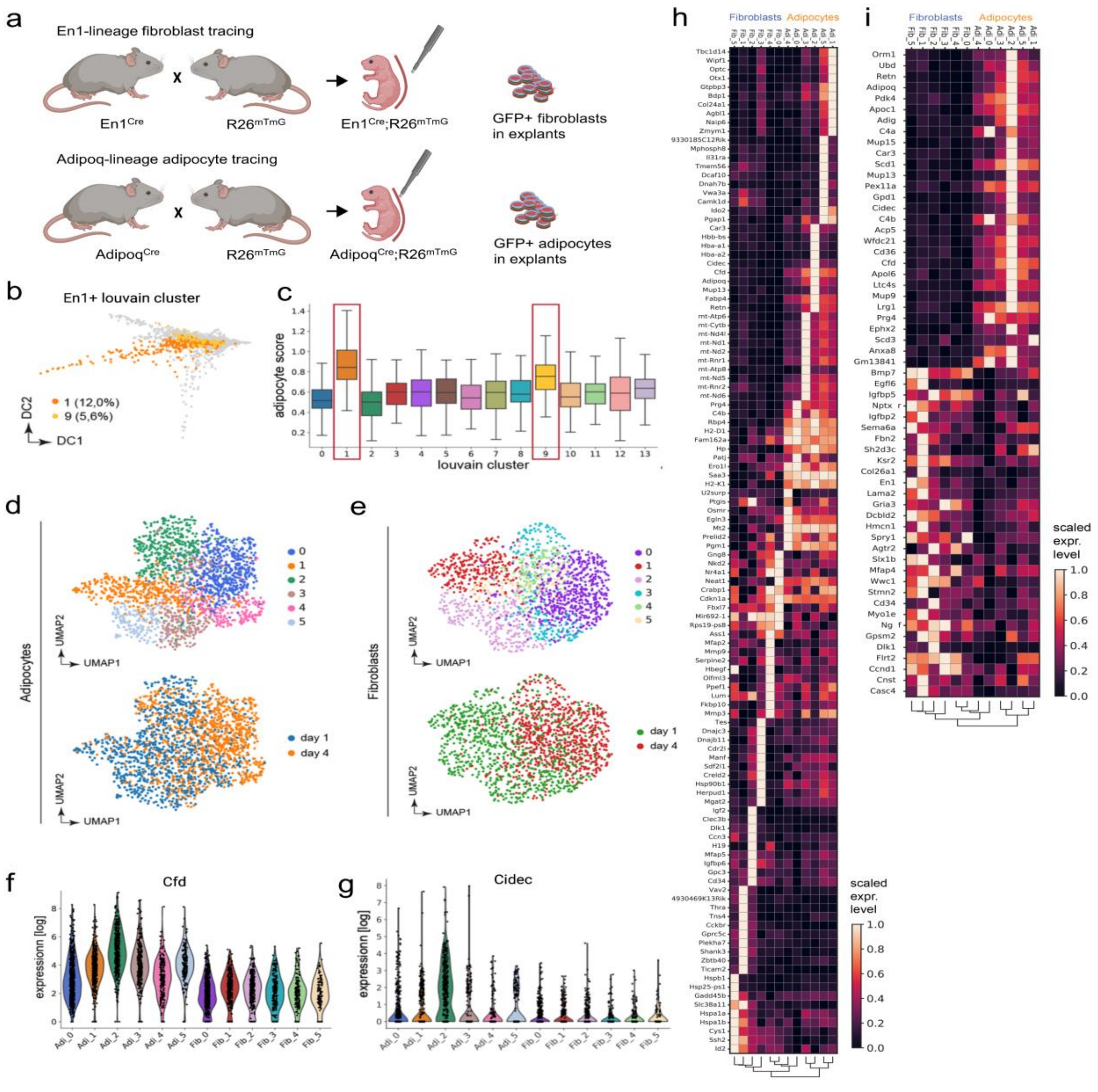


**Supplementary Figure 1. Adipocytes retain their identity in skin explant model.**

**a**, Scheme of adipocyte- and fibroblast-lineage tracing approach in transgenic mice. **b**, **c**, En1-lineage positive stromal cells in neonatal skin used for explants contains two clusters of adipocyte progenitors, cluster 1 and 9. En1 grouping at day 1 and day 4 of scar explant assay: scoring of adipocyte gene signature from MCA neonatal skin on En1 data; Louvain clustering was used to identify adipocyte-enriched clusters (**b**). En1 Louvain clustering with adipocyte expression on cluster 1 and 9; adjacent is a box plot displaying adipocyte score in specific cluster (**c**). The adipocyte progenitors are

excluded in the analyses of mature adipocytes. n = 2000 cells, the centre lines indicate the median, the lower and upper boundaries of the boxes mark the first and third quartile, and the lower and upper boundaries of the whiskers mark the minimum and maximum. **d**, Six distinct sub clusters of cells in Adipoq$^{Cre}$ lineage across Day1 and Day4. **e**, Six distinct sub clusters of cells in En1$^{Cre}$ across day 1 and day 4. **f**, **g**, Expression of mature adipocyte genes Cfd and Cidec with comparable expression in migratory and mature adipocytes, with marginal expression across all fibroblast clusters. **h**, Heatmap showing top 10 marker genes in each of the 12 cell clusters across fibroblast and adipocyte lineages. **i**, Heatmap showing core signature genes distinguishing adipocytes from fibroblasts.

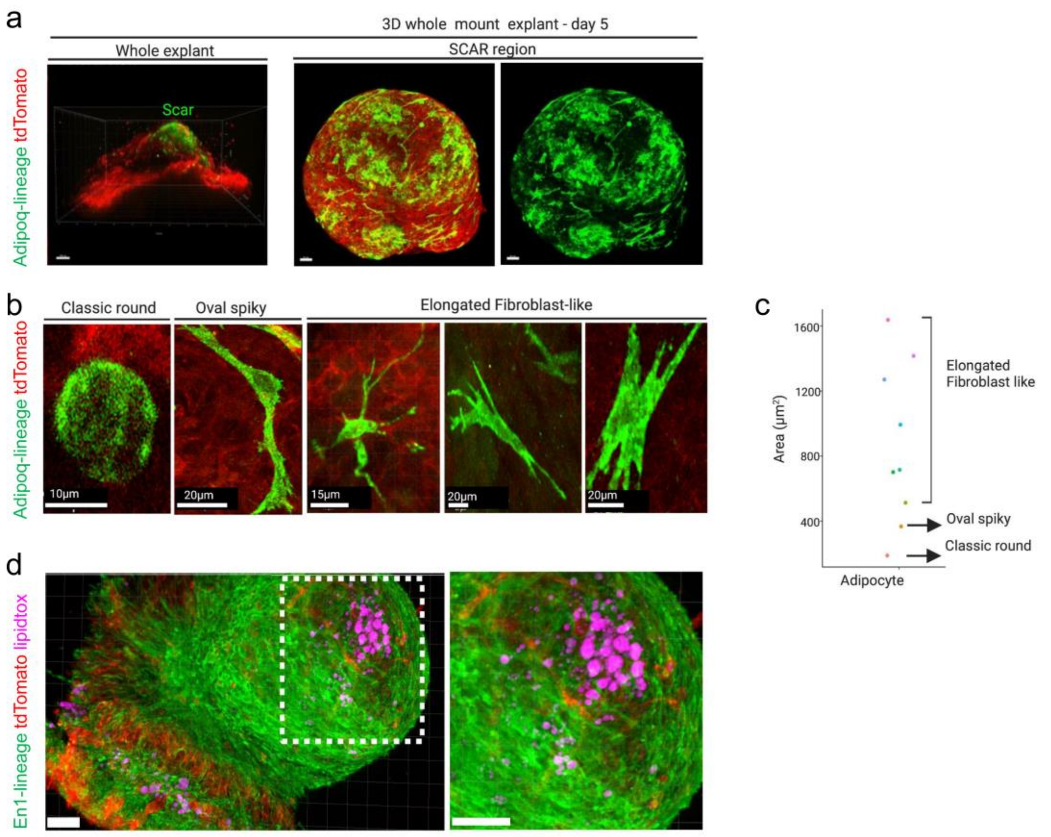


**Supplementary Figure 2. Adipocytes attain migratory morphologies, and revert to rounded cells.**

**a**, 3D view of Adipoq$^{Cre}$;R26$^{mTmG}$ skin explant showing adiponectin-lineage cells (GFP) that migrated into the formative scar tissue. scale bar: 100µm. Merged (left) and Adipoq-lineage (GFP) (right). Scale bar: 50 µm. **b**, Diverse morphologies of adiponectin-lineage cells (GFP) cells seen in Adipoq$^{Cre}$;R26$^{mTmG}$ explants. Representative examples of each type of morphology. This experiment was repeated three times independently with similar results. **c**, Size quantification of varying morphologies of adipocyte-lineage cells. **d**, Lipid tox staining showing mature round lipid-filled adipocytes on top of scar tissue in a day 5 En1$^{Cre}$;R26$^{mTmG}$ explant. En1-lineage fibroblasts (GFP) are negative for lipid tox. Scale bars: 70 µm. This experiment was repeated three times independently with similar results.

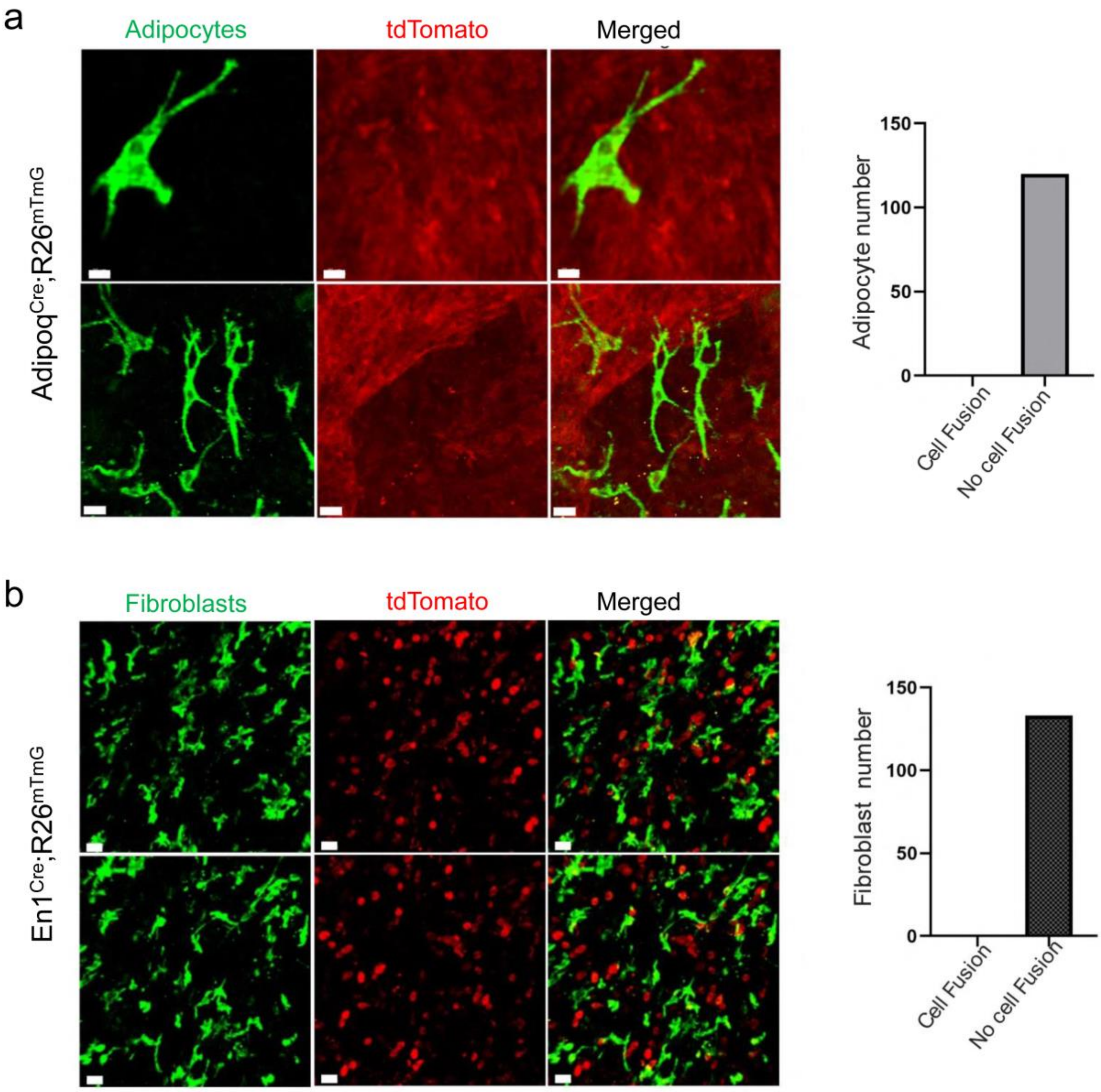


**Supplementary Figure 3. Lack of cell fusions between adipocyte and fibroblast lineages.**

**a**, GFP+ adipocytes were analysed for cell fusion events at single cell resolution in explants from Adipoq$^{Cre}$;R26$^{mTmG}$ and there was no cell fusion with tdTomato+ fibroblasts. **b**, GFP+ fibroblasts were analysed for cell fusion events at single cell resolution in explants En1$^{Cre}$;R26$^{mTmG}$ showing no cell fusion with tdTomato+ cells and other lineage negative cells. Scale bars: 20 µm. These experiments were repeated three times independently with similar results.

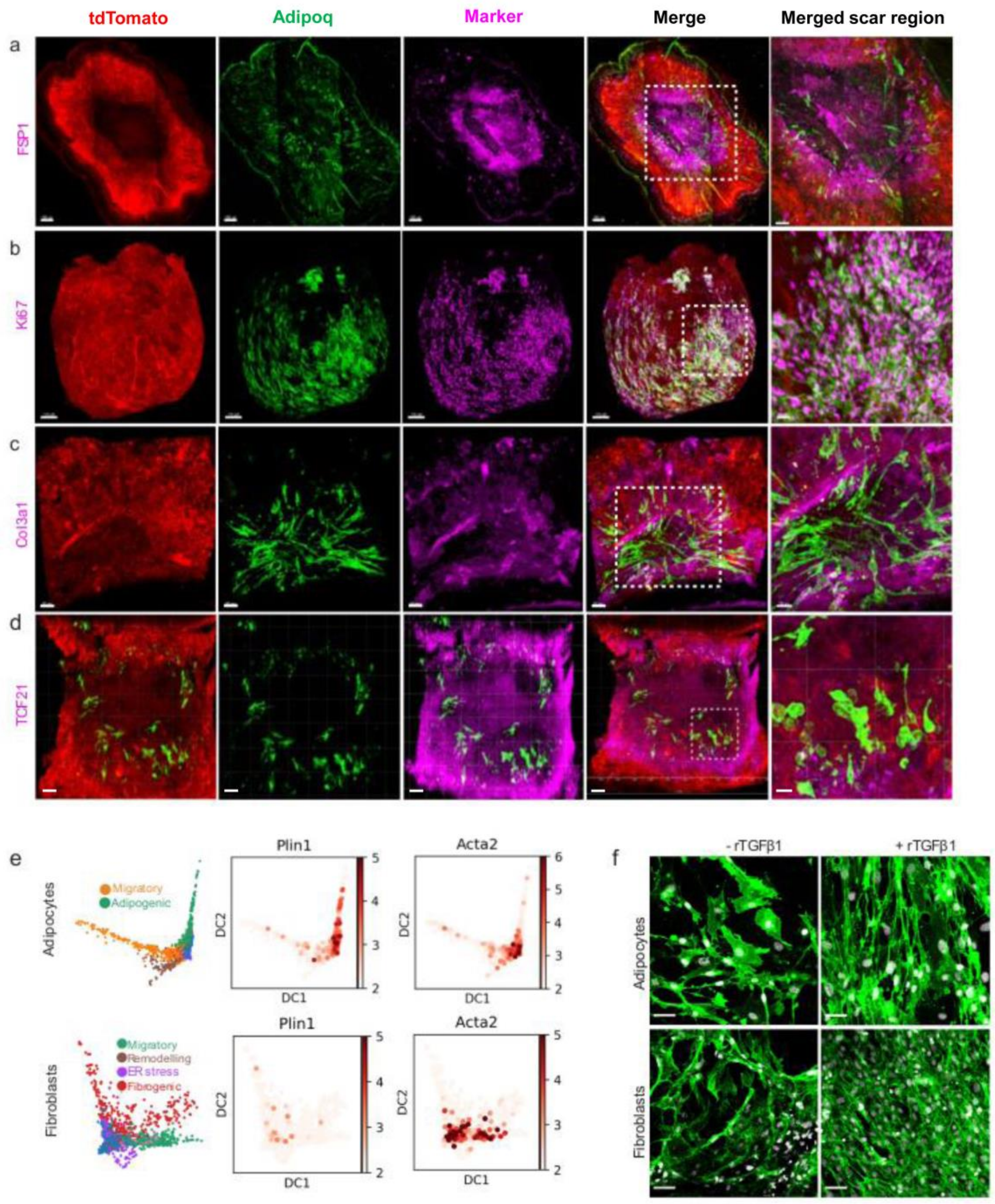


**Supplementary Figure 4. Migratory adipocytes in ex vivo conditions do not express classical myofibroblast markers despite morphological changes. a-d**, 3D Immunostainings performed on Adipoq$^{+}$GFP$^{+}$ *ex vivo* explant tissue on day 4 and day 5. Migratory adipocytes do not express the fibroblast markers fibroblast specific protein 1 (FSP1), extracellular matrix Col3a1, and TCF21. Scale bars: 100 µm in low magnification images, 20 µm in high magnification images. **e**, Dynamic expression of

adipocyte marker Perilipin1 and myofibroblast marker Acta2 (αSMA). In adipocytes, Perilipin1 is higher in the adipogenic states and Acta2 is low in both adipogenic and migratory states. In contrast, in fibroblasts, Perilipin1 is low in both states and Acta2 is higher in the migratory state of the fibroblasts. **f**, Adipocytes acquire migratory morphologies treatment with recombinant TGFβ1. Scale bars: 50 μm. These experiments were repeated three times independently with similar results.

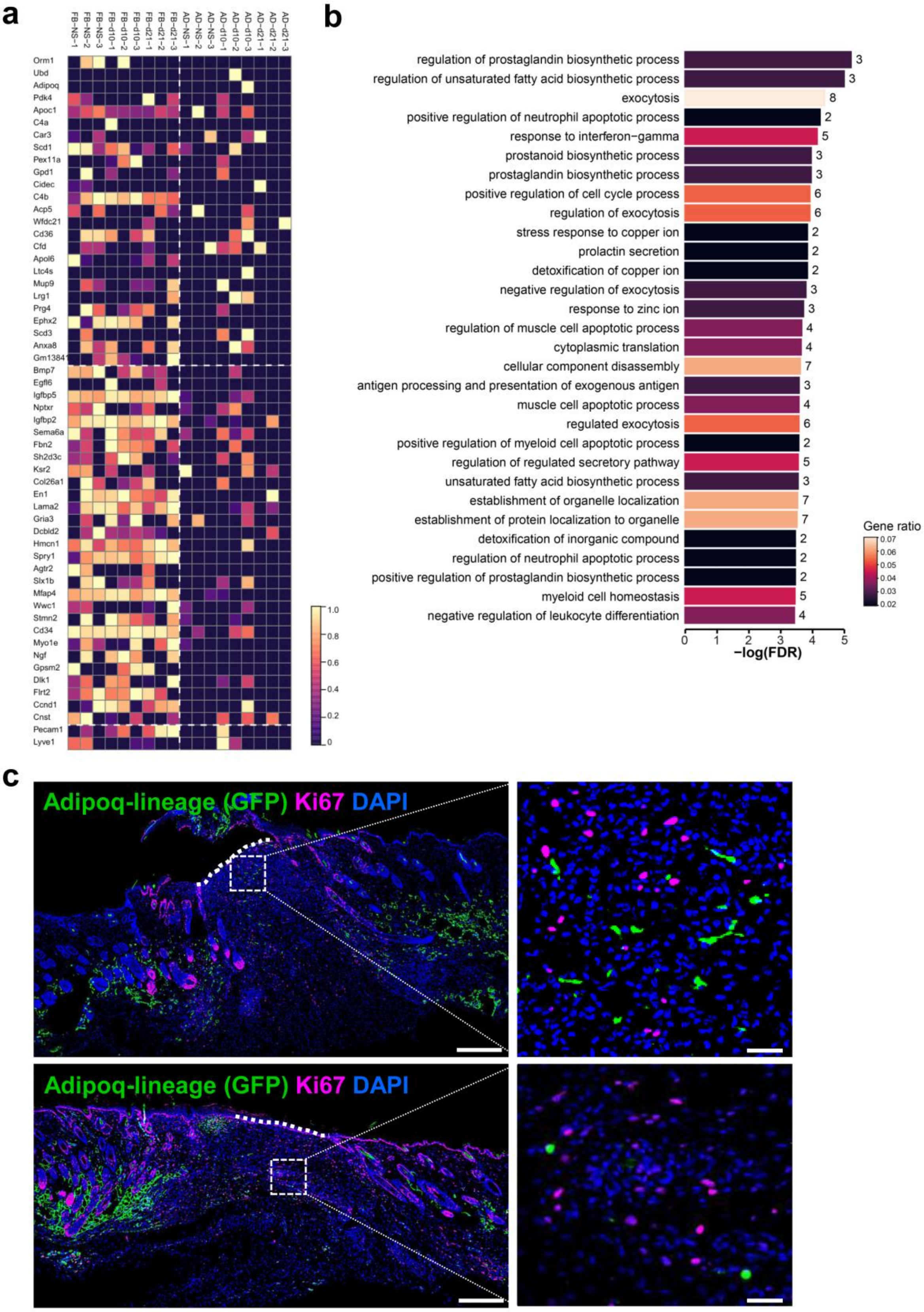


**Supplementary Figure 5. Adipocytes are non fibrogenic in wounds.**

a, Heatmap shows scaled gene expression of adipocytes core signature gene and fibroblast core signature gene expression in FACS sorted adipocytes and fibroblasts from day 7 and 21 wounds and adjacent skin of Adipoq$^{Cre}$;R26$^{mTmG}$ and En1$^{Cre}$;R26$^{mTmG}$ mice, respectively. b, GO term enrichment based on DGEs in adipocytes from day 7, day 21 wounds and adjacent skin. Filled colour represented number of genes enriched relative all different expressed genes compared to adipocyte from uninjured skin. Each cell type at each time point includes three independent biological replicates. c, Immunostaining of Ki67 on day 7 (upper panel) and day 21 (lower panel) wounds from Adipoq$^{Cre}$;R26$^{mTmG}$ mice. The dotted lines indicate the wound regions, the dotted boxes indicate the regions that are shown in high magnification images. Scale bars: 100 µm in low magnification images, 10 µm in high magnification images. These experiments were performed with three independent samples with similar results.
