# Supplementary material for: Wound infiltrating adipocytes are not myofibroblasts
Source: Nat Commun. 2023 May 25;14:3020. doi: 10.1038/s41467-023-38591-6 (PMC10213017; doi:10.1038/s41467-023-38591-6)
Supplement: Supplementary file 2 — Description of Additional Supplementary Files [file 41467_2023_38591_MOESM2_ESM.pdf]

Description of Additional Supplementary Files

Supplementary Movie 1. Live imaging of day 0 skin explant obtained from *Adipoq*^{Cre};*R26*^{mTmG} mice.

Supplementary Movie 2. Live imaging of day 1 skin explant obtained from *Adipoq*^{Cre};*R26*^{mTmG} mice.

Supplementary Movie 3. Live imaging of day 2 skin explant obtained from *Adipoq*^{Cre};*R26*^{mTmG} mice.

Supplementary Movie 4. Live imaging of day 3 skin explant obtained from *Adipoq*^{Cre};*R26*^{mTmG} mice.

Supplementary Movie 5. Live imaging of day 4 skin explant obtained from *Adipoq*^{Cre};*R26*^{mTmG} mice.

Supplementary Movie 6. Live imaging of day 5 skin explant obtained from *Adipoq*^{Cre};*R26*^{mTmG} mice.

Supplementary Movie 7. Migratory tracks of adipocytes in live imaging of mCherry nuclear reporter (*Adipoq*^{Cre};*R26*$^{LSL\text{-}H2B\text{-}mCherry}$) skin explant from 24 h to 48 h. The colour indicates the time, with blue at the beginning and red at the end of the video.

Supplementary Movie 8. Migratory tracks of fibroblasts in live imaging of mCherry nuclear reporter (*En1*^{Cre};*R26*$^{LSL\text{-}H2B\text{-}mCherry}$) skin explant from 24 h to 48 h. The colour indicates the time, with blue at the beginning and red at the end of the video.

Supplementary Movie 9. Migratory tracks of adipocytes in live imaging of mCherry nuclear reporter (*Adipoq*^{Cre};*R26*$^{LSL\text{-}H2B\text{-}mCherry}$) skin explant from 96 h to 120 h. The colour indicates the time, with blue at the beginning and red at the end of the video.

Supplementary Movie 10. Migratory tracks of fibroblasts in live imaging of mCherry nuclear reporter (*En1*^{Cre};*R26*$^{LSL\text{-}H2B\text{-}mCherry}$) skin explant from 96 h to 120 h. The colour indicates the time, with blue at the beginning and red at the end of the video.

Supplementary Data 1. List of differentially expressed genes detected in 12 clusters generated from mcSCRB-seq dataset. Differential expression was determined by diffxpy with two-side Wald test with Benjamini-Hochberg correction. Gene ontology (GO) classification of each cluster was enriched with two-side Fisher's exact test with Benjamini-Hochberg corrections using GOATOOLS.

Supplementary Data 2. List of differentially expressed signature genes of adipocytes and fibroblasts in the mcSCRB-seq dataset, and the gene ontology (GO) classification of each cell type using gene set enrichment analysis (GSEA). Differential expression was determined by diffxpy with two-side Wald test with Benjamini-Hochberg correction. GO classification of each cluster was enriched with two-side Fisher's exact test with Benjamini-Hochberg corrections using GOATOOLS.
